# Supplementary figures and images for: Cohesin is required for meiotic spindle assembly independent of its role in cohesion in C. elegans
Source: PLoS Genet. 2022 Oct 24;18(10):e1010136. doi: 10.1371/journal.pgen.1010136 (PMC9632809; doi:10.1371/journal.pgen.1010136)

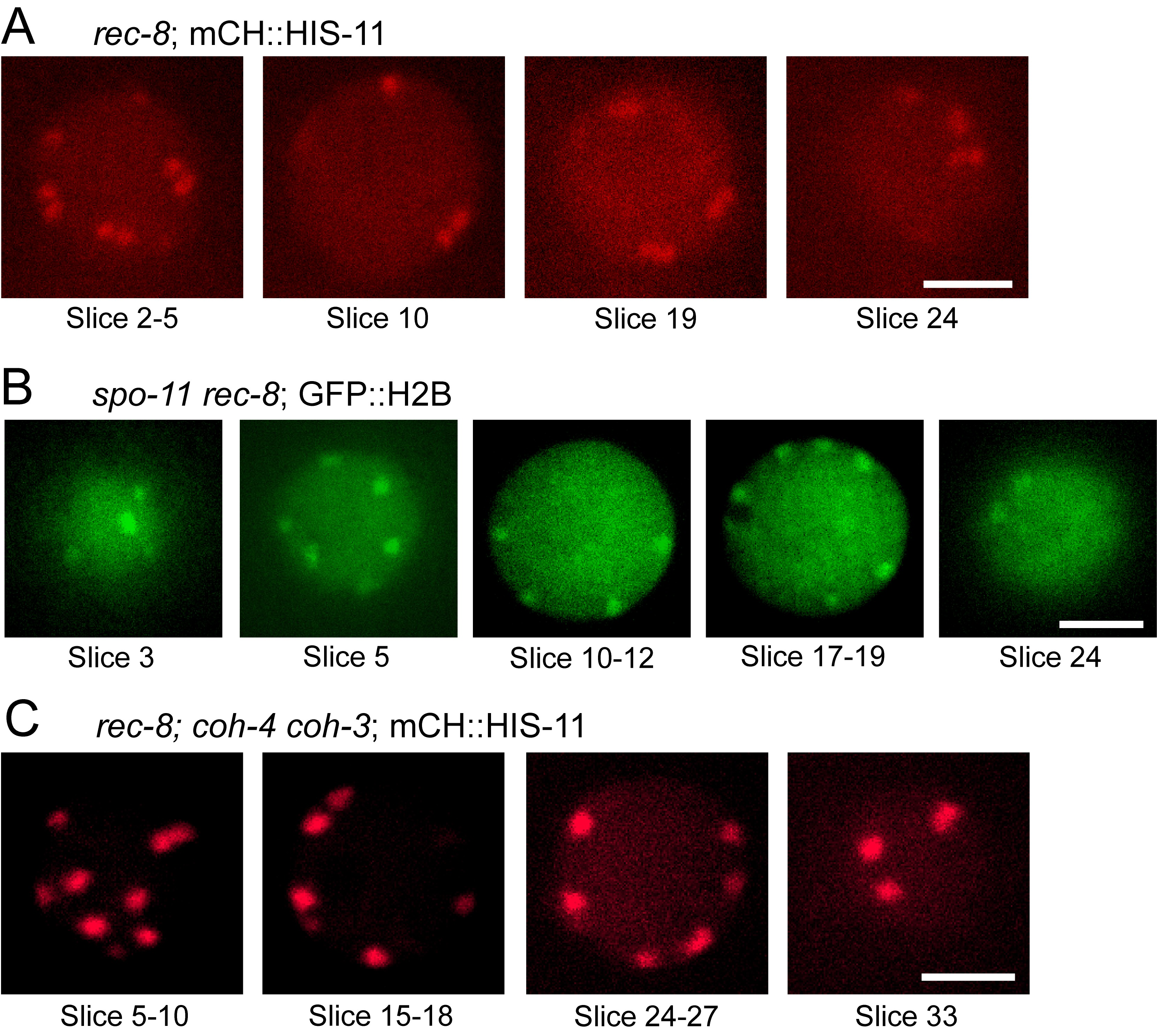

Supplement: S1 Fig — (A) Single and Z-stack sum slices of a living rec-8 oocyte nucleus expressing mCherry::HIS-11. rec-8 oocyte nuclei contained 12.33 +/- 0.37 DNA bodies (n = 9), which included univalents and an occasional chromatid. (B) Single and Z-stack sum slices of a living spo-11 rec-8 oocyte nucleus expressing GFP::H2B show 22 of the 24 total chromatids. spo-11 rec-8 oocyte nuclei contained 23.8 +/- 0.01 DNA bodies (n = 10). (C) Single and Z-stack sum slices of a living rec-8; coh-4 coh-3 oocyte nucleus expressing mCH::HIS-11. rec-8; coh-4 coh-3 nuclei contained 24.5 +/- 0.43 DNA bodies (n = 14). 4/14 oocytes contained one or two small DNA bodies which may indicate chromosomes fragmented by SPO-11 activity. All bars = 5 μm. (TIF) [file pgen.1010136.s001.tif]

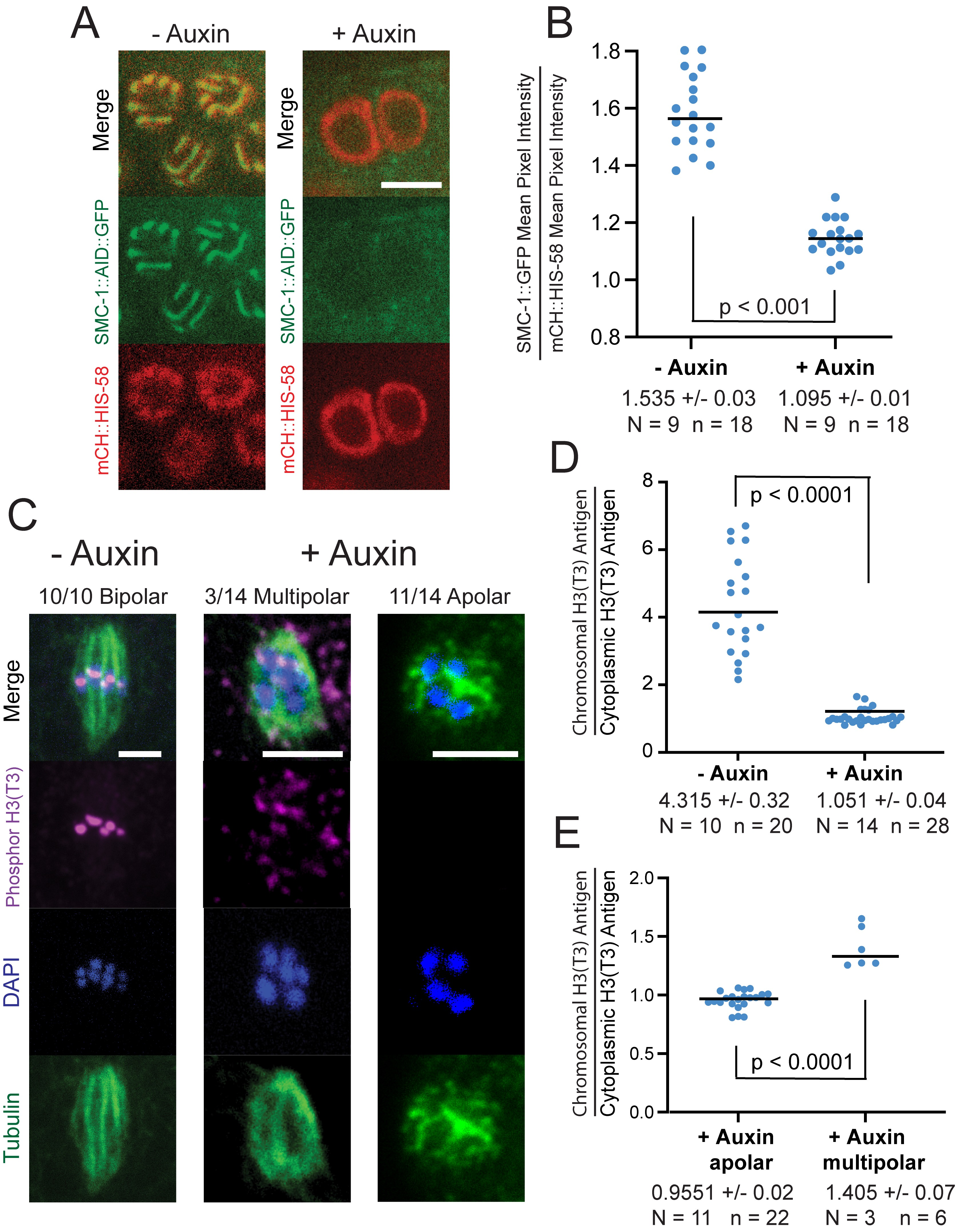

Supplement: S2 Fig — (A) Single-plane images of SMC-1::AID:GFP in the gonad of living worms incubated overnight in either the presence or absence of auxin. (B) The ratio of SMC-1::AID::GFP mean pixel intensity to mCH:HIS-58 mean pixel intensity in gonad nuclei was determined in worms incubated as described in (A). Several of the ratios in auxin-treated worms approach the values obtained in untreated worms. N, number of worms. n, number of nuclei. (C) Embryos from worms expressing HASP-1::AID and incubated in either the presence or absence of auxin were fixed and stained with tubulin and phosphor H3(T3) antibodies, and with DAPI. (D) Ratios of chromosomal to cytoplasmic H3(T3) antibody staining were determed in worms incubated as described in (C). N, number of spindles. n, number of chromosomes. (E) The values for worms incubated in the presence of auxin were separated into those obtained from chromosomes in apolar spindles and those obtained from chromosomes in multipolar spindles. N, number of spindles. n, number of chromosomes. All bars equal 4μm. (TIF) [file pgen.1010136.s002.tif]

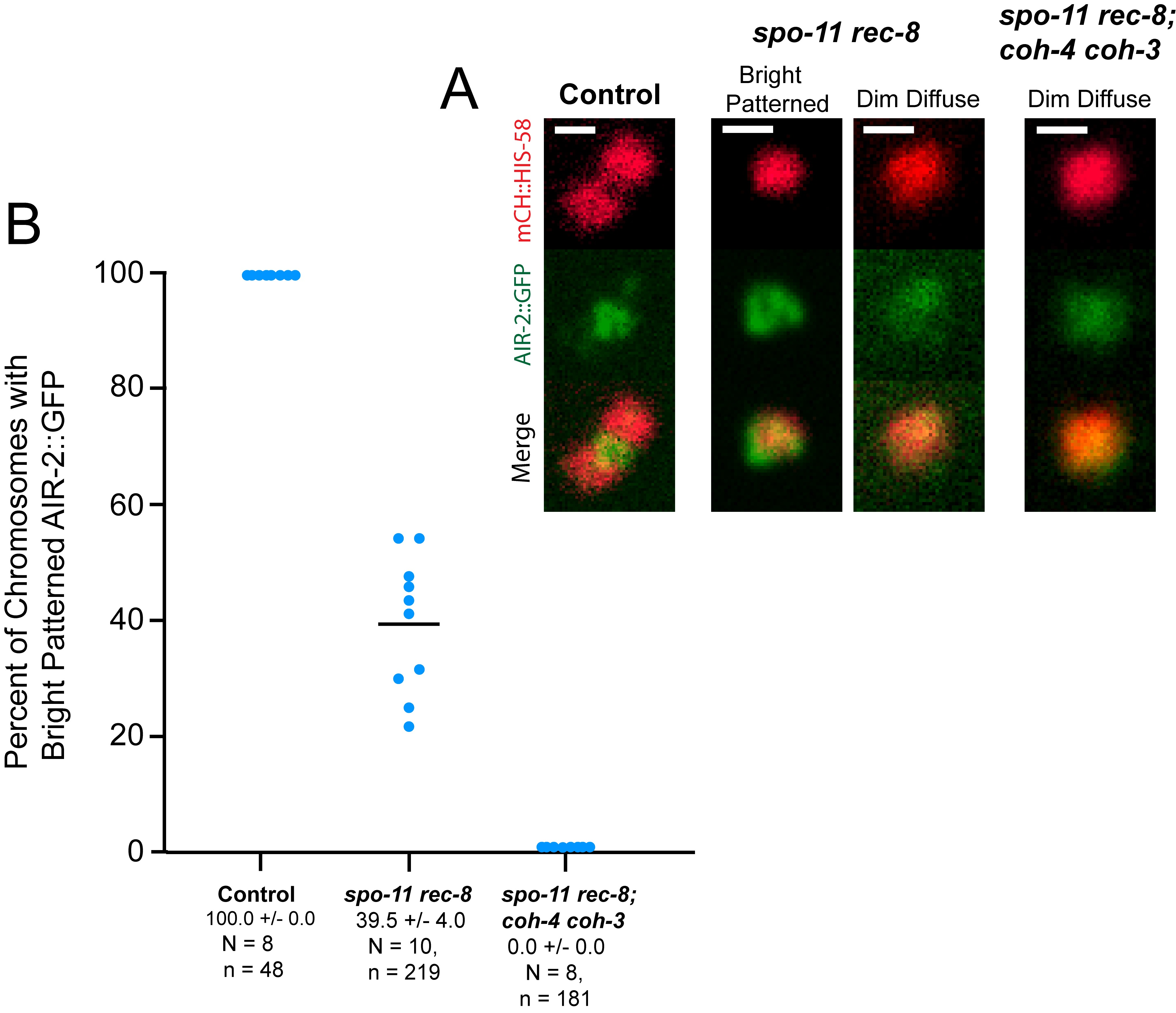

Supplement: S3 Fig — (A) Single chromosomes from z-stack images of living control and mutant C. elegans oocytes expressing mCherry::HIS-58 and AIR-2::GFP. Two examples are shown of a spo-11 rec-8 chromosome, one bound by bright patterned AIR-2::GFP and one with dim diffuse AIR-2::GFP. All bars = 1μm. (B) Graph showing the percent of chromosomes bound by bright AIR-2::GFP in living -1 oocytes of control and mutant C. elegans. Z-stacks of entire nuclei were analyzed. For spo-11 rec-8, bright vs dim AIR-2::GFP was scored by only comparing chromatids within the same focal plane. Bright AIR-2::GFP was observed on 100 percent of control chromosomes, 0 percent of spo-11 rec-8; coh-4 coh-3 chromatids and 39.5 +/- 4.0 percent of spo-11 rec-8 chromatids. N, number of oocytes. n, number of chromosomes. (TIF) [file pgen.1010136.s003.tif]

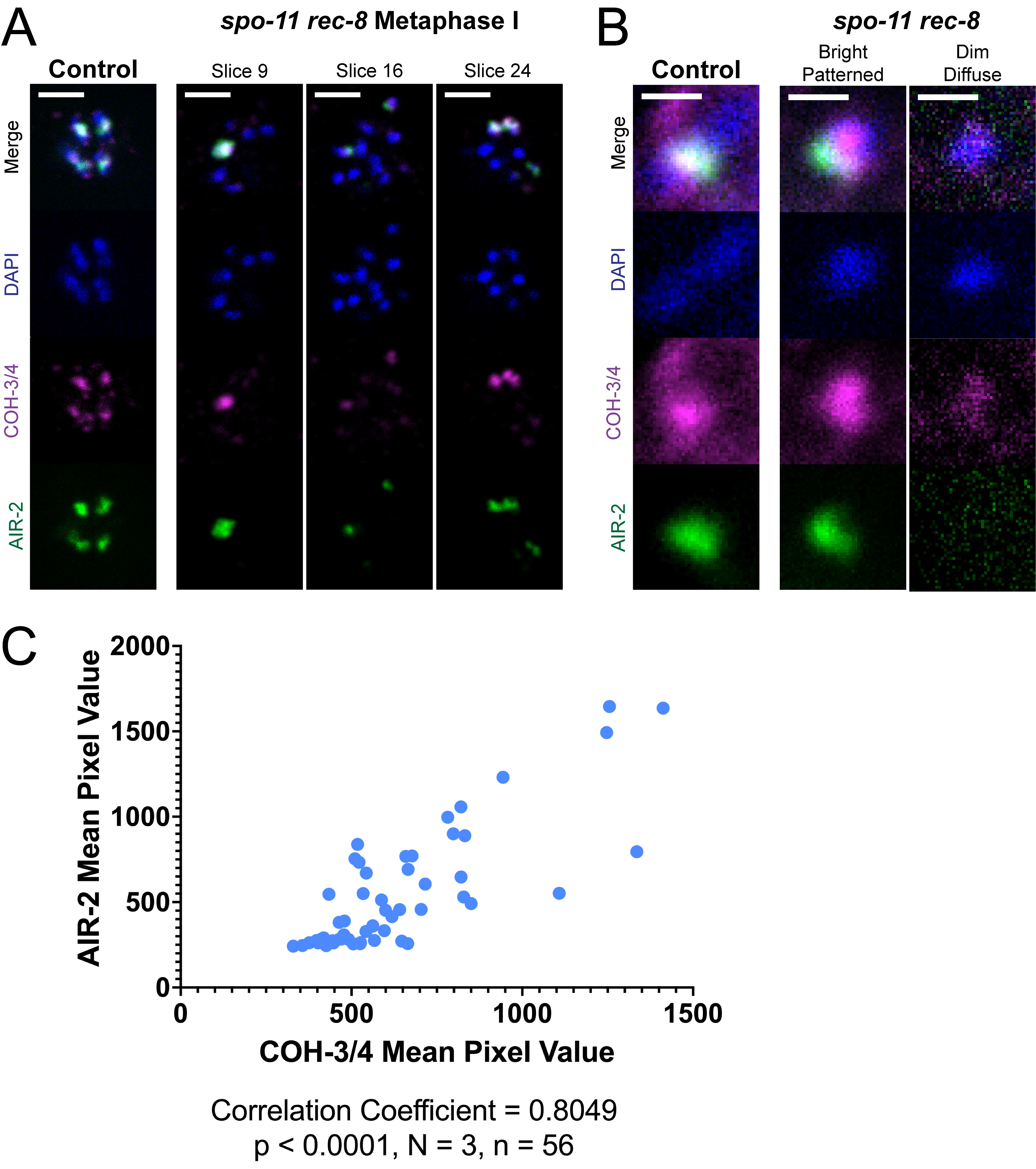

Supplement: S4 Fig — (A) Meiotic embryos within control and spo-11 rec-8 worms expressing AIR-2::GFP were fixed and stained with DAPI, COH-3/4 antibodies, and GFP antibodies. The control spindle displays consistent intensities of AIR-2 and COH-3/4 on each chromosome while the spo-11 rec-8 spindle displays varying intensities. Bars = 3 μm. (B) High magnification view of single chromatids from (A). The control chromosome shows bright COH-3/4 and bright AIR-2. Two chromosomes from the same spo-11 rec-8 embryo are shown, one with bright COH-3/4 and AIR-2 and one with dim COH-3/4 and AIR-2. Bars = 1 μm. (C) Graph showing mean pixel value of COH-3/4 versus mean pixel value of AIR-2 on rec-8 spo-11 chromosomes. Mean pixel values were taken by using a circle ROI with a 22 pixel diameter (covering the entire univalent’s area). N, number of embryos. n, number of chromosomes. (TIF) [file pgen.1010136.s004.tif]

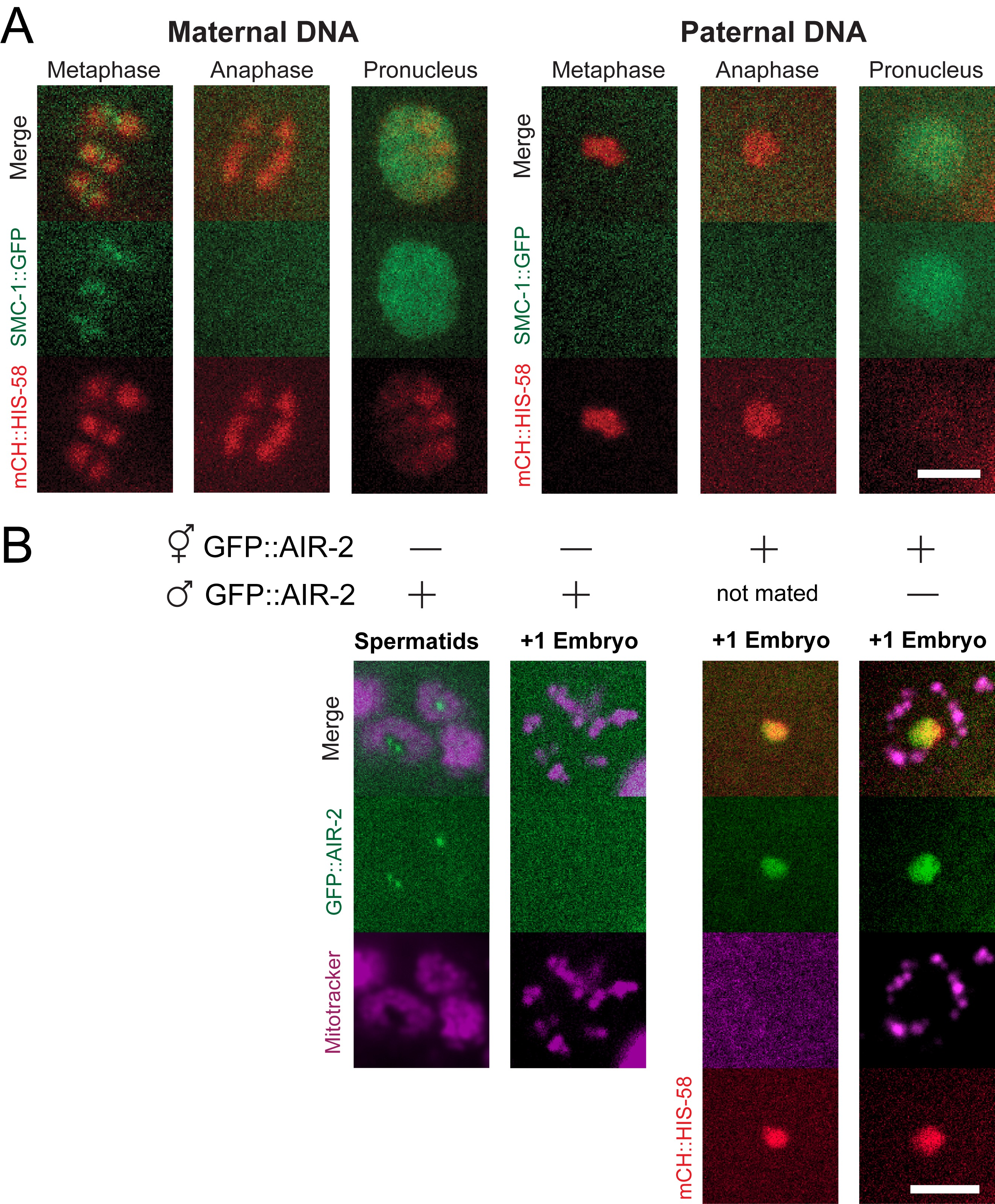

Supplement: S5 Fig — (A) Time-lapse images of 15/15 embryos from worms expressing SMC-1::GFP and mCH::HIS-58 in both oocytes and spermatocytes show no SMC-1::GFP on sperm-derived paternal DNA within the zygote during meiosis. SMC-1::GFP was observed in the sperm-derived paternal pronucleus in 7/7 embryos. Bar = 3 μm. (B) Male worms were soaked in mitotracker before mating to hermaphrodites. The sperm-derived paternal DNA is found at the center of the cloud of paternal mitochondria within meiotic embryos (far right). In 5/5 mated hermaphrodites, paternal AIR-2::GFP was present on spermatids, but was not detected post-fertilization within the cloud of paternal mitochondria in meiotic embryos identified by their position in the uterus adjacent to the spermatheca (+1 embryo). 13/13 unmated hermaphrodites expressing AIR-2::GFP, and 11/11 AIR-2::GFP expressing hermaphrodites mated with non-expressing males had AIR-2::GFP on the sperm DNA in +1 embryos. Bar = 4μm. (TIF) [file pgen.1010136.s005.tif]

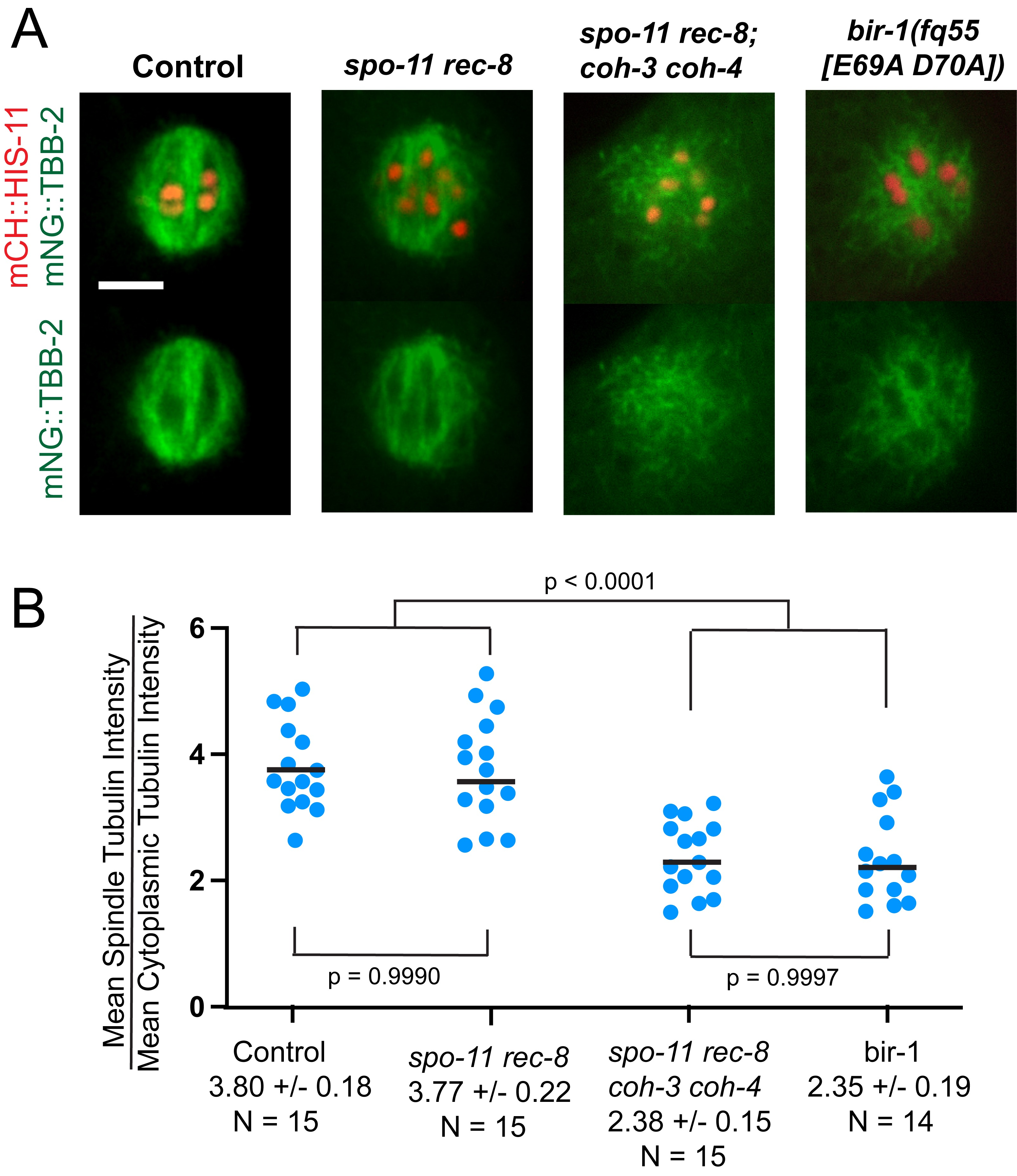

Supplement: S6 Fig — (A) Single slices from z-stack images of embryos expressing mNG::TBB-2 and mCH::HIS-11. Bar = 4μm. (B) Ratios of mean, background-subtracted mNG::TBB-2 pixel values in spindles vs. nearby cytoplasm of control and mutant embryos. N = number of embryos. (TIF) [file pgen.1010136.s006.tif]

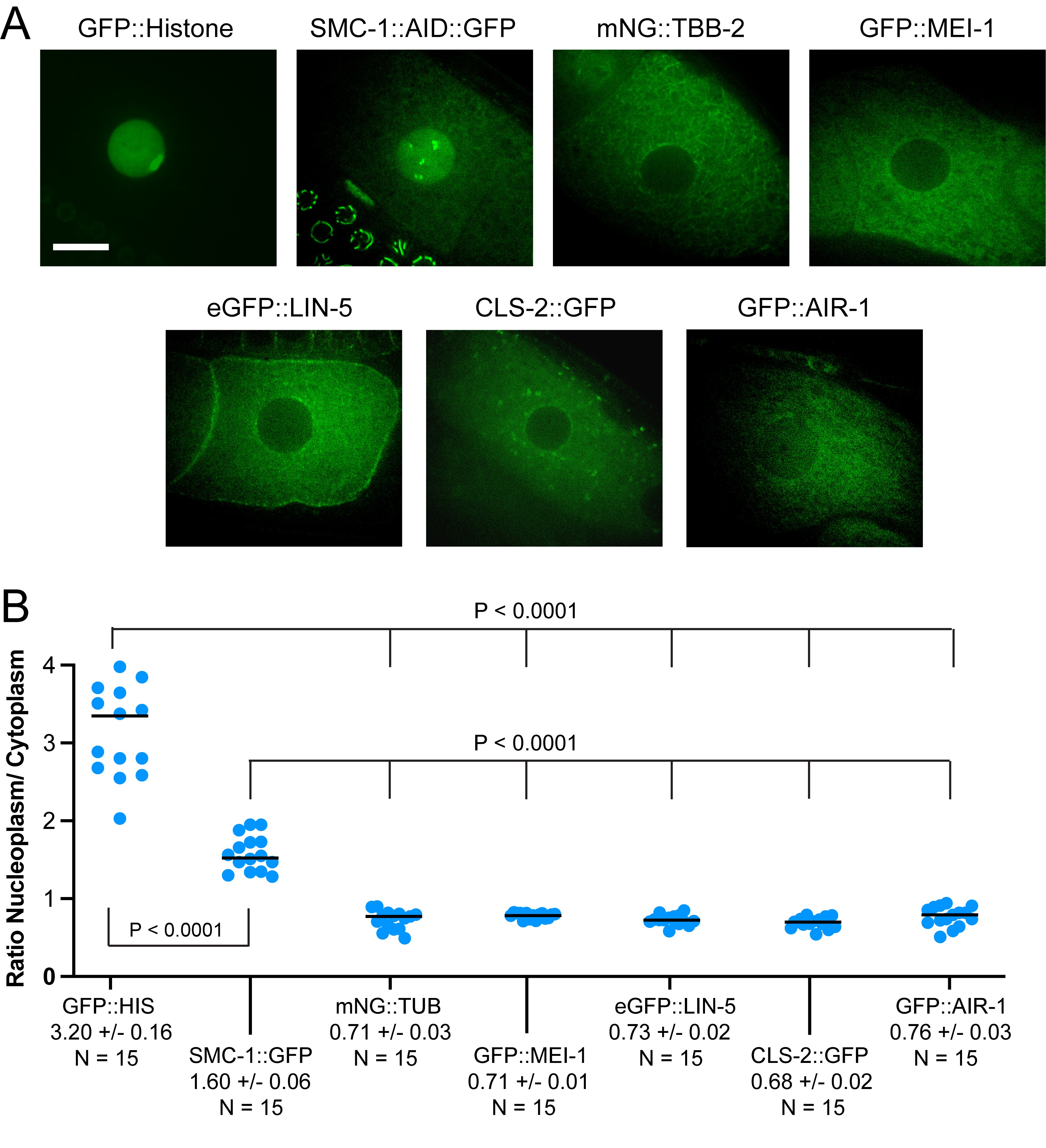

Supplement: S7 Fig — (A) Single plane images of -1 oocytes in C. elegans expressing GFP::H2B, SMC-1::AID::GFP, and spindle assembly factors. Bar = 10 μm. (B) Nucleoplasmic to cytoplasmic ratios were determined for mean, background-subtracted pixel values in -1 oocytes. (TIF) [file pgen.1010136.s007.tif]

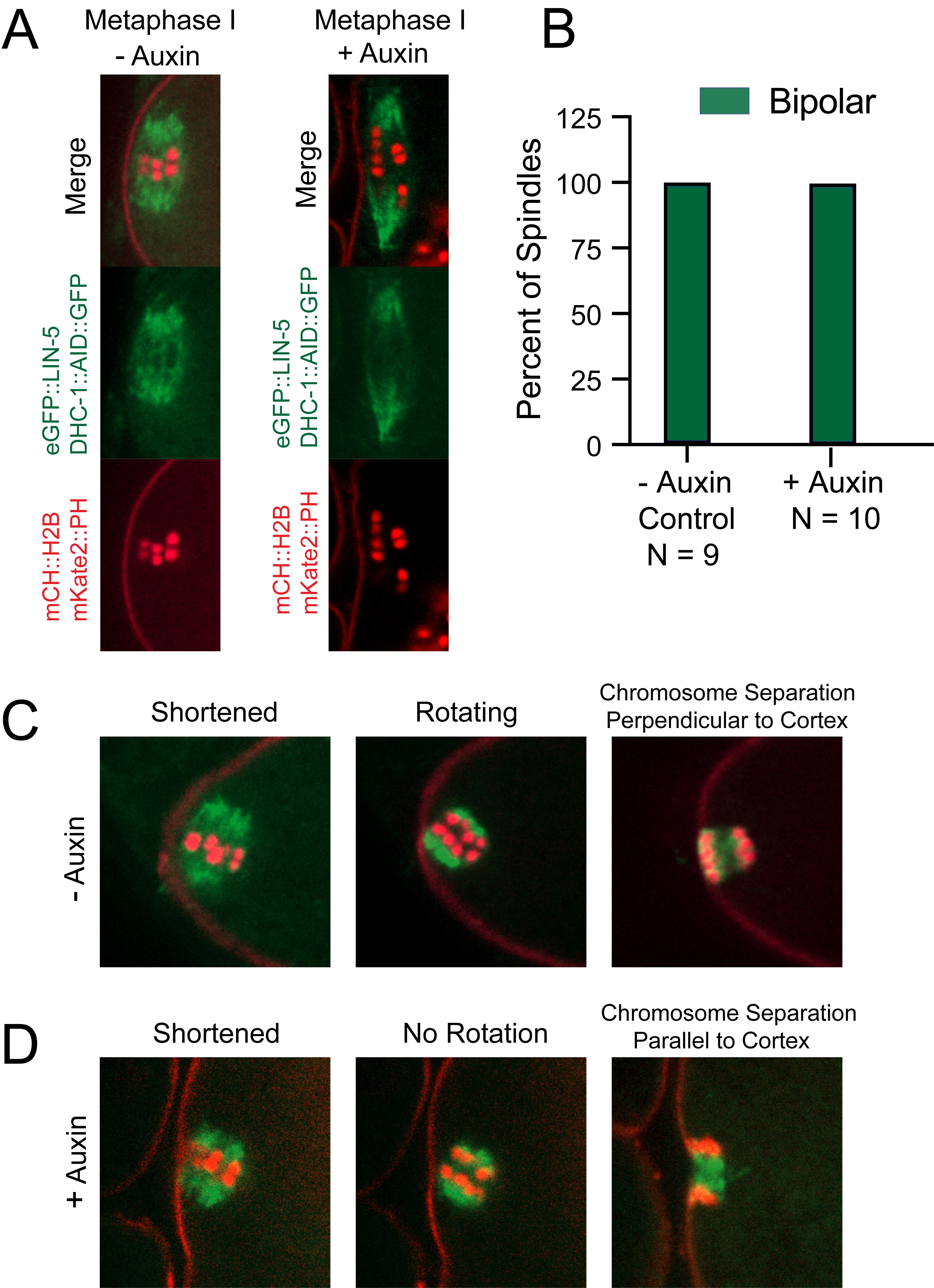

Supplement: S8 Fig — C. elegans expressing DHC-1::AID::GFP, eGFP::LIN-5, mCH::H2B and mKate2::PH were incubated for 2–4 hours in the presence or absence of auxin. (A) Images of metaphase I spindles show that 9/9 spindles were bipolar in the absence of auxin and 10/10 metaphase I spindles were bipolar in the presence of auxin. (B) Quantification of spindle bipolarity. (C) Time-lapse images of C. elegans incubated in the absence of auxin show bipolar spindles shorten and rotate prior to chromosome separation (n = 5). (D) Time-lapse images of C. elegans incubated in the presence of auxin show bipolar spindles shorten and remain parallel to the cortex due to the depletion of DHC-1::AID::GFP (n = 7). (TIF) [file pgen.1010136.s008.tif]
