# Supplementary material for: Cohesin is required for meiotic spindle assembly independent of its role in cohesion in C. elegans
Source: PLoS Genet. 2022 Oct 24;18(10):e1010136. doi: 10.1371/journal.pgen.1010136 (PMC9632809; doi:10.1371/journal.pgen.1010136)
Supplement: S1 Table — (DOCX) [file pgen.1010136.s009.docx]

**S1 Table. Hatch rate data for auxin-induced degron experiments**

|  |  | Unhatched eggs | Hatched larvae # (%) |
| --- | --- | --- | --- |
| *hasp-1(AID) tir1 GFP::lin-5 mKate::tubulin mCherry::H2b mKate::PH*  FM712 | ethanol | 0 | 685 (100%) |
|  | auxin | 98 | 35 (26%) |
| *hasp-1(AID) tir1 GFP::AIR-2 mcherry::h2b*  FM720 | ethanol | 0 | 680 (100%) |
|  | auxin | 183 | 36 (16%) |
| *smc-1(AID) tir1 GFP::lin-5 mKate::tubulin*  FM743 | ethanol | 5 | 730 (99%) |
|  | auxin | 157 | 1 (0.6%) |
| *GFP::lin-5*  FM400 | ethanol | 4 | 856 (99%) |
|  | auxin | 4 | 695 (99%) |
| *dhc-1(AID) tir1 GFP::lin-5 mcherry::h2b mKate::PH*  FM485 | ethanol | 12 | 436 (97%) |
|  | auxin | 0 | 1 (sterile) |
| *air-2(AID) tir1 GFP::lin-5 mcherry::h2b mKate::PH*  FM729 | ethanol | 32 | 875 (96%) |
|  | auxin | 326 | 0 (0%) |

A *hasp-1(tm3858)* deletion strain is 100% sterile with mitotic germline defects. A different *hasp-1(AID)* has 25% hatching and phosphor-H3(T3) staining in the distal gonad indicating partial depletion [1].

*smc-1* null alleles, *him-1(h55)* and *him-1(h134)* are sterile [2] or lethal [3].

**REFERENCES**

1. Macaraeg J, Reinhard I, Ward M, Carmeci D, Stanaway M, Moore A, et al. Genetic analysis of C. elegans Haspin-like genes shows that hasp-1 plays multiple roles in the germline. Biol Open. 2022 Jul 15;11(7):bio059277. doi: 10.1242/bio.059277.

2. Chan RC, Chan A, Jeon M, Wu TF, Pasqualone D, Rougvie AE, et al. Chromosome cohesion is regulated by a clock gene paralogue TIM-1. Nature. 2003; 423: 1002-9. doi:10.1038/nature01697.

3. Howell A, Gilmour S, Mancebo R, Rose A. Genetic analysis of a large autosomal region in Caenorhabditis elegans by the use of a free duplication. Genetical Research. 1987; 49(3): 207-213. doi:10.1017/S0016672300027099.
