# Supplementary material for: Cohesin is required for meiotic spindle assembly independent of its role in cohesion in C. elegans
Source: PLoS Genet. 2022 Oct 24;18(10):e1010136. doi: 10.1371/journal.pgen.1010136 (PMC9632809; doi:10.1371/journal.pgen.1010136)
Supplement: S2 Table — List of genotypes of all strains used in this paper. (DOCX) [file pgen.1010136.s010.docx]

**S2 Table. *C. elegans* Strain List**

ATG368 *bir-1(fq55[E69A D70A])/ nT1[unc-?(n754) let-?(qIs50)] (IV;V)*

ATG385 *hasp-1(fq52 [hasp-1::degron]) I; ieSi38 [Psun-1p::TIR1::mRuby::sun1 3’UTR + Cbr-unc-119(+)] IV* . Degron described in Ferrandiz et al., 2018.

ATG415 *smc-1(fq64[smc-1::AID::GFP]) I; ieSi38 [Psun-1p::TIR1::mRuby::sun-1 3'UTR + Cbr-unc-119(+)] IV.* Degron described in Castellano-Pozo et al., 2020.

BN452 *bqSi189 [lmn-1p::mCherry::his-58 + unc-119(+)] II; mel-28(bq5[GFP::mel-28]) III*

EU2875 *mei-1(or1937[GFP::mei-1]) I; ltIs37 IV*

EU2876 *aspm-1(or1935[GFP::aspm-1]) I; ltIs37 IV*

FGP103 *ieSi65 [sun-1p::TIR1::sun-1 3’UTR + cb-unc-119(+)] II;*

*cls-2(syb819[cls-2::degron::GFP]) unc-119(ed3) III; ltIs37 IV.* Degron described in Pelisch et al., 2019.

FGP118 *air-2(syb418[degron::wrmScarlet::air-2]) I; ieSi65 [sun-1p::TIR1::sun-1 3’UTR + Cbr-unc-119(+)] II; ruIs32 [pie-1::GFP::histone + cb-unc-119]
unc-119(ed3) III.* Degron described in Pelisch et al., 2019.

FM97 *him-8(e1489) ltIs37 IV*

LP447 *klp-7(cp178[klp-7::mNG-C1::3xFlag]) III*

OD142 *ltIs78 [(pK05) pie-1::GFP::TEV::Stag::air-1 spliced coding + unc-119(+)]*

OD3230 *air-2(lt58[air2::GFP::tev::loxP::3xFlag]) I*

OD4362 *ltSi1412 [pNA20; Pmex-5::mNeonGreen::tbb-2 operon linker mCh::his-11::Ptbb-2 + cb-unc-119(+)] I; unc-119(ed3) III*

TY5124 *spo-11(me44) rec-8(ok978) / nT1 IV; coh-4(tm1857) coh-3(gk112) / nT1[qIs51] V*

VC666 *rec-8(ok978) / nT1[qIs51] IV*

FM597 *ruIs32 [pie-1p::GFP::H2B + unc-119(+)] III; spo-11(me44)/ nT1[qIs51] IV; wjIs76[Cn_unc-119(+); pie-1p::mKate2::tba-2]*

FM666 *ltSi1412 [pNA20; Pmex-5::mNeonGreen::tbb-2 operon linker mCh::his-11::Ptbb-2 + Cbr-unc-119(+)] I; rec-8(ok978) / nT1[qIs51] IV*

FM667 *bqSi189 [lmn-1p::mCherry::his-58 + unc-119(+)] II; mel-28(bq5[GFP::mel-28]) III; rec-8(ok978) / nT1[qIs51] IV*

FM681 *air-2(It58[air-2::GFP::tev::loxP::3XFlag]) I; bqSi189 [lmn-1p::mCherry::his-58 + unc-119(+)] II; rec-8(ok978) / nT1[qIs51] IV*

FM683 *aspm-1(or1935 [GFP::aspm-1]) I; rec-8(ok978) / nT1[qIs51] IV*

FM663 *ltSi1412 [pNA20; Pmex-5::mNeonGreen::tbb-2 operon linker mCh::his-11::Ptbb-2; Cbr-unc-119(+)] I; spo-11(me44) rec-8(ok978) / nT1 IV; coh-4(tm1857) coh-3(gk112) / nT1[qIs51] V*

FM684 *air-2(It58[air-2::GFP::tev::loxP::3XFlag]) I; bqSi189 [lmn-1p::mCherry::his-58 + unc-119(+)] II; spo-11(me44) rec-8(ok978) / nT1 IV; coh-4(tm1857) coh-3(gk112) / nT1[qIs51] V*

FM671 *ruIs32 [pie-1p::GFP::H2B + unc-119(+)] III; spo-11(me44) rec-8(ok978) / nT1[qIs51] IV; wjIs76[Cn_unc-119(+); pie-1p::mKate2::tba-2]*

FM682 *air-2(It58[air-2::GFP::tev::loxP::3XFlag]) I; bqSi189 [lmn-1p::mCherry::his-58 + unc-119(+)] II; spo-11(me44) rec-8(ok978) / nT1[qIs51] IV*

FM701 *mei-1(or1937[GFP::mei-1]) I; bqSi189 [lmn-1p::mCherry::his-58 + unc-119(+)] II; spo-11(me44) rec-8(ok978) / nT1[qIs51] IV*

FM754 *smc-1(fq64[smc-1::AID::GFP]) I; bqSi189 [lmn-1p::mCherry::his-58 + unc-119(+)] II; spo-11(me44) rec-8(ok978) / nT1[qIs51] IV* Degron described in Castellano-Pozo et al., 2020.

FM773 *ltSi1412 [pNA20; Pmex-5::mNeonGreen::tbb-2 operon linker mCh::his-11::Ptbb-2; Cbr-unc-119(+)] I; spo-11(me44) rec-8(ok978) / nT1[qIs51] IV*

FM774 *bqSi189 [lmn-1p::mCherry::his-58 + unc-119(+)] II; cls-2(syb819[cls-2::degron::GFP]) III; spo-11(me44) rec-8(ok978) / nT1[qIs51] IV* Degron described in Pelisch et al., 2019.

FM798 *bqSi189 [lmn-1p::mCherry::his-58 + unc-119(+)] II; klp-7(cp178[klp-7::mNG-C1::3xFlag]) III; spo-11(me44) rec-8(ok978) / nT1[qIs51] IV*

FM799 *air-2(syb418[degron::wrmScarlet::air-2]) I; klp-7(cp178[klp-7::mNG-C1::3xFlag]) III; spo-11(me44) rec-8(ok978) / nT1[qIs51] IV* Degron described in Pelisch et al., 2019.

FM801 *air-2(syb418[degron::wrmScarlet::air-2]) I; cls-2(syb819[cls-2::degron::GFP]) III; spo-11(me44) rec-8(ok978) / nT1[qIs51] IV* Degron described in Pelisch et al., 2019.

FM702 *ltSi1412 [pNA20; Pmex-5::mNeonGreen::tbb-2 operon linker mCh::his-11::Ptbb-2 + Cbr-unc-119(+)] I; bir-1(fq55 [E69A D70A]) / nT1[qIs51] V*

FM707 *air-2(It58[air-2::GFP::tev::loxP::3XFlag]) I; bqSi189 [lmn-1p::mCherry::his-58 + unc-119(+)] II; bir-1(fq55 [E69A D70A]) / nT1[qIs51] V*

FM775 *bqSi189 [lmn-1p::mCherry::his-58 + unc-119(+)] II; klp-7(cp178[klp-7::mNG-C1::3xFlag]) III; bir-1(fq55[E69A D70A]) / nT1[qIs51] V*

FM743 *smc-1 (fq64[smc-1::AID::GFP]) I, lin-5(he244[egfp::lin-5] II; ieSi38 [sun-1p::TIR1::mRuby::sun-1 3'UTR + Cbr-unc-119(+)] IV, wjIs76[Cn_unc-119(+); pie-1p::mKate2::tba-2]* Degron described in Castellano-Pozo et al., 2020.

FM712 *hasp-1(fq52 [hasp-1::degron]) I, lin-5(he244[egfp::lin-5] II; duSi10 [mex-5p::mCh::H2B operon linker mKate2::PH inserted in K03H6.5] ieSi38 [sun-1p::TIR1::mRuby::sun-1 3'UTR + Cbr-unc-119(+)] IV; wjIs76[Cn_unc-119(+); pie-1p::mKate2::tba-2].* Degron described in Ferrandiz et al., 2018.

FM720 *hasp-1(fq52 [hasp-1::degron]) I; cpIs103[ Psun-1>TIR1-C1::F2A::mTagBFP2-C1::NLS] II, ltIs37 [pie-1p::mCherry::H2B::pie-1 3'UTR + unc-119(+)] IV; ltIs14 [(pASM05) pie-1p::GFP-TEV-STag::air-2 + unc-119(+)].* Degron described in Ferrandiz et al., 2018.

FM750 *fxIs1 [pie-1p::TIR1::mRuby] air-2(ie31[degron::GFP::AIR-2]) I; him-5(e1490) V*

FM642 *air-2(lt58[air2::GFP::tev::loxP::3xFlag]) I;* *him-8(e1489) ltIs37 IV*

FM507 *wjIs76[Cn_unc-119(+); pie-1p::mKate2::tba-2]*

TY5121 *rec-8(ok978)/nT1 IV; coh-4(tm1857) coh-3(gk112) V/nT1 [qIs51] V*.

FM944 *rec-8(ok978)/nT1 IV; coh-4(tm1857) coh-3(gk112)/nT1[qIs51] V; ltSi1412*

*[pNA20; Pmex-5::mNeonGreen::tbb-2 operon linker mCh::his-11::Ptbb-2;*

*cb-unc-199(+)]I; unc-119(ed3)III (MOS I insertion)*

FM945 *air-2(It58[air-2::GFP::tev::loxP::3XFlag]) I; bqSi189 [lmn-1p::mCherry::his-*

*58 + unc-119(+)] II; rec-8(ok978)/nT1 IV; coh-4(tm1857) coh-3*

*(gk112)/nT1[qIs51] V*
