# Supplementary material for: Cohesin is required for meiotic spindle assembly independent of its role in cohesion in C. elegans
Source: PLoS Genet. 2022 Oct 24;18(10):e1010136. doi: 10.1371/journal.pgen.1010136 (PMC9632809; doi:10.1371/journal.pgen.1010136)
Supplement: Attachment — Submitted filename: rebuttal.rtf [file pgen.1010136.s018.rtf]

Dear Dr McNally,

Thank you very much for submitting your Research Article entitled 'Cohesin is required for meiotic spindle assembly independent of its role in cohesion in C. elegans' to PLOS Genetics.
The manuscript was fully evaluated at the editorial level and by independent peer reviewers. The reviewers appreciated the attention to an important problem, but raised some substantial concerns about the current manuscript. Based on the reviews, we will not be able to accept this version of the manuscript, but we would be willing to review a much-revised version. We cannot, of course, promise publication at that time.

Should you decide to revise the manuscript for further consideration here, your revisions should address the specific points made by each reviewer. We will also require a detailed list of your responses to the review comments and a description of the changes you have made in the manuscript.
For resubmission I ask you to carefully address all reviewer's comments. The reviewers are very clear in their comments as to which experiments are required, and which changes and additions to the text are requested.  This includes not only performing the required experiments and text edits, but also addressing low n values and missing information in the materials and methods section. I think it is possible to address these concerns in the timeframe suggested for revisions.

If you decide to revise the manuscript for further consideration at PLOS Genetics, please aim to resubmit within the next 60 days, unless it will take extra time to address the concerns of the reviewers, in which case we would appreciate an expected resubmission date by email to plosgenetics@plos.org.

If present, accompanying reviewer attachments are included with this email; please notify the journal office if any appear to be missing. They will also be available for download from the link below. You can use this link to log into the system when you are ready to submit a revised version, having first consulted our Submission Checklist.

To enhance the reproducibility of your results, we recommend that you deposit your laboratory protocols in protocols.io, where a protocol can be assigned its own identifier (DOI) such that it can be cited independently in the future. Additionally, PLOS ONE offers an option to publish peer-reviewed clinical study protocols. Read more information on sharing protocols at https://plos.org/protocols?utm_medium=editorial-email&utm_source=authorletters&utm_campaign=protocols

Please be aware that our data availability policy requires that all numerical data underlying graphs or summary statistics are included with the submission, and you will need to provide this upon resubmission if not already present. In addition, we do not permit the inclusion of phrases such as "data not shown" or "unpublished results" in manuscripts. All points should be backed up by data provided with the submission.

While revising your submission, please upload your figure files to the Preflight Analysis and Conversion Engine (PACE) digital diagnostic tool.  PACE helps ensure that figures meet PLOS requirements. To use PACE, you must first register as a user. Then, login and navigate to the UPLOAD tab, where you will find detailed instructions on how to use the tool. If you encounter any issues or have any questions when using PACE, please email us at figures@plos.org.

PLOS has incorporated Similarity Check, powered by iThenticate, into its journal-wide submission system in order to screen submitted content for originality before publication. Each PLOS journal undertakes screening on a proportion of submitted articles. You will be contacted if needed following the screening process.

To resubmit, use the link below and 'Revise Submission' in the 'Submissions Needing Revision' folder.


We are sorry that we cannot be more positive about your manuscript at this stage. Please do not hesitate to contact us if you have any concerns or questions.

Yours sincerely,

Sarit Smolikove
Guest Editor
PLOS Genetics

Gregory P. Copenhaver
Editor-in-Chief
PLOS Genetics


Reviewer's Responses to Questions
Comments to the Authors:
Please note here if the review is uploaded as an attachment.
Reviewer #1: Although cohesin is best known for its role in the sister chromatid cohesion (SCC) that holds replicated sister chromatids together from S phase until anaphase of mitosis and meiosis, the cohesin complex appears to perform other roles that are independent of its role in SCC. This manuscript from Frank McNally's lab reports a novel SCC-independent role for cohesin in the establishment of bipolar, acentrosomal meiotic spindles. In many organisms, including C. elegans and mammals, oocytes lack centrosomes, and chromosomes appear to play a critical role in the formation of meiotic spindles. How spindle bipolarity is established in the absence of centrosomes remains poorly understood, but it has seemed likely that the back-to-back, bipolar arrangement of homologous chromosomes in meiosis I and of sister chromatids in meiosis II might be critical. By comparing spindle assembly in fertilized oocytes from C. elegans hermaphrodites that lack meiotic cohesin with those from mutants in which cohesin associates with meiotic chromosomes but does mediate SCC, the authors demonstrate that cohesin, but not SCC, is required for spindle bipolarity. As long as COH-3/4 cohesin is present on chromosomes, robust, bipolar spindles can form even when pairs of sister chromatids are detached from one another, indicating that back-to-back orientation of chromosomes is not needed for spindle bipolarity. Instead, COH-3/4 cohesin appears to recruit the Aurora B kinase AIR-2 to meiotic chromosomes through mechanism dependent on the Haspin kinase and survivin, and AIR-2 in turn appears to regulate the chromosomal association of several factors known to function in oocyte spindle assembly. Thus, this paper overturns a widely-held assumption regarding how spindle bipolarity is achieved, at least in C. elegans, provides new mechanistic insight into the process, and demonstrates a novel SCC-independent role for cohesin.

The experimental approach exclusively involves live imaging of microtubule and chromosome dynamics, as well as fluorescently-tagged versions of various microtubule and chromosome-associated proteins. When possible, quantitative data were extracted from the images. Although the methods used for the quantitation and certain technical aspects of the study need to be better described, the major conclusions are convincing and quite interesting. Some suggestions that may strengthen the conclusions or improve the readability of the paper follow.

Major Comments:
1. p.5, lines 19-20 and elsewhere: the term “single chromatid” is confusing. Phrases like “mutants that start meiotic spindle assembly with single chromatids” sound like there is only one chromatid. It might be clearer to say “detached sister chromatids” or “separated sister chromatids.” We have changed all “single chromatids” to “separated sister chromatids” with the exception of the discussion of HeLa cells with unreplicated chromatids.
2. Chromosome separation was not observed during anaphase I or II of spo-11 rec-8; coh-4 coh-3 quadruple mutants (p.8, lines 5-7 and 8-10), but it was observed during meiosis I of spo-11 rec-8 double mutants (p.8, lines 18-20). Since the authors likely have the data, it would be interesting to know whether polar body extrusion ever occurred in the quadruple mutant, and how often it occurred during meiosis I and II in the double mutant.  We have added the statement that polar bodies did not form in 10/10 time-lapse sequences of the quadruple mutant and cite the single polar body reported by Severson and Meyer 2009 for the double mutant.
3. p.8, lines 21-23: “At metaphase II” is confusing. The mass of microtubules presumably assembled before metaphase II, while the failure to separate chromatids happened after metaphase II. We have changed “At metaphase II” to “During meiosis II”.
4. p.10, lines 1-4: “This non-cohesive COH-3/4 cohesin might be removed by separase at anaphase I, leaving the metaphase II chromatids with no cohesin. This hypothesis was validated by time-lapse imaging…” The imaging validated the hypothesis that cohesin was undetectable on metaphase II chromatids, but not that separase was required for removal of the COH-3/4 cohesin. This is likely true, but the authors should be careful not to suggest that it is proven by this experiment.  We have removed the words “by separase”.
5. p.11, line 11 and throughout: The terms “spindle-incompetent” and “spindle-competent” are confusing if not misleading. Apolar spindles form around the detached sister chromatids of spo-11 rec-8; coh-4 coh-3 mutants (abstract line 10, first subheading in the results section, p.6 line 19, etc.). Thus, the mutants described as spindle-incompetent do not fail to form a meiotic spindle, they just do not form a bipolar spindle.  We have changed these adjectives to “bipolar-spindle-competent” and “bipolar-spindle-incompetent”.
6. p.12, lines 2-4 and Fig. 4 D-F: in spo-11 rec-8 double mutants, some chromatids have very bright AIR-2 signal and others do not. Since COH-3/4 protein, but not SCC, appears to be required for the bright AIR-2, it would be quite interesting to know if the chromatids with intense AIR-2 signal also have high levels of COH-3/4.  We attempted this experiment with COH-3::mCherry and GFP::AIR-2, however, the COH-3 fusion was too dim for this analysis.
7. pp.12-13: bir-1(E69A, D70A) mutants enter meiosis with 6 bivalents but form apolar spindles, and chromosomes do not separate in meiosis I or II. Is SCC released and do sisters disjoin in anaphase I or II in these mutants? If so, this should be shown. If not, this should also be shown, since it would increase the impact of this paper - SCC likely persists due to a failure to recruit AIR-2 to the region between homologs in meiosis I and between sisters in meiosis II, and therefore a failure to phosphorylate REC-8 to mark it for destruction by separase. Thus, the failure to segregate chromosomes could occur for very different reasons in the bir-1 mutant and in the spo-11 rec-8; coh-4 coh-3 mutants. From the figures, it does appear that disjunction fails, and this is quite intersting and critical to interpret the data. We agree that the reason for anaphase failure in the bir-1 mutant and the cohesin mutants might be different because of the well-established role of AIR-2 in phosphorylating REC-8 to allow separase cleavage.  However, the focus of this manuscript is bipolar spindle assembly rather than the role of the CPC in cohesin release at anaphase.  Thus were are not adding more discussion that might distract the reader from the main point of the paper.
  8. p.14, lines 14-16: “These results suggested that cohesin-dependent AIR-2 regulates proteins that coalesce microtubule bundles and promote microtubule polymerization.” This model is consistent with the data, but it is not the only model consistent with the data. Thus, the statement as written is too strong.  The sentence has been replaced with :”These results suggested that cohesin-dependent AIR-2 might regulate proteins that promote coalescence of microtubule bundles and promote microtubule polymerization, although other models are possible.”
9. p.14, lines 19-22: It is difficult to understand the hypothesis, since activating MT-binding proteins required for coalescence of MT bundles or MT polymerization and inhibiting such proteins should result in opposite phenotypes. The hypothesis has been restated to take this into account.
10. pp.16-17: the final section of the results does not fit with the rest of the paper and does not add much to the story. It is not obvious to me how the experiments “address the relative roles of the ran pathway and the CPC pathway,” as is claimed. Indeed, presence of SAFs in the cytoplasm does not preclude their regulation by Ran. Canonical targets of the ran pathway have NLSs that are released from importin by GTP-ran.  Thus it is unlikely that a protein without an NLS would be activated by the canonical ran pathway.  We have clarified this rationale and softened the conclusion.  The section should be rewritten to clarify the rationale and avoid overstating the conclusions, or perhaps the authors may wish to remove it since doing so would not significantly weaken the paper.
11. p.17, lines 19-21: It does not seem likely that the removal of residual cohesin at anaphase II would prevent the formation of a metaphase III spindle between meiosis II and the first mitotic cell cycle, because spindles DO form in the absence of cohesin during meiosis I and II of spo-11 rec-8; coh-4 coh-3 mutants – they just remain apolar. The word “bipolar” has been added.
12. As written, the manuscript suggests that the SCC-independent role of cohesin in bipolar meiotic spindle assembly is unique to the acentrosomal spindles that form in oocytes. This is likely true; however, it is formally possible that cohesin also plays an SCC-independent role that is required for formation of the centriolar meiotic spindles that form during spermatogenesis. It would be easy to look at spindle formation during spermatogenesis in spo-11 rec-8 and bir-1(E69A, D70A) mutants compared to spo-11 rec-8; coh-4 coh-3 mutants to see of loss of SCC or loss of cohesin disrupts spindle polarity. At the very least, this should be addressed in the discussion.  We have added the following sentence to the discussion: “In addition, future analysis of centrosome-based C. elegans male meiosis in cohesin mutants should reveal whether the cohesin-dependence of spindle bipolarity is specific to acentrosomal spindle assembly.”    
13. p.18: the long discussion paragraph on this page jumps from topic to topic and is somewhat difficult to follow. The possibility that Aurora B inhibits a target SAF comes out of the blue. After several examples of Aurora B-dependent inhibition of SAFs, the model is raised that the CPC might affect spindle pole assembly through activation or inhibition of multiple SAFs. Are there known examples of activation? The bir-1 mutants fails to accumulate KLP-7 in midbivalent rings and fails to exclude CLS-2 from a similar region, so there are clearly effects on protein localization – is this what the authors mean by activation and inhibition, or are they referring to enzymatic activities? The authors cite published data showing that depletion of either protein results in multipolar spindles and mention that effects of overexpression are not known. It is difficult to disentangle this data and understand what the model is. The paragraph needs to be revised, and a model figure would be very helpful.  This paragraph has been re-organized by splitting into separate paragraphs about activation, inhibition, and altered localization of SAFs.
14. p.18, lines 22-23 and p.19 lines 1-4: the fact that metaphase I defects were not observed following SMC3 depletion in Drosophila means that one cannot conclude that the bipolar spindle assembly observed in Drosophila sunn mutants suggests an SCC-independent requirement for cohesin in spindle assembly. We did not and do not suggest this. It could simply be that cohesin is not required for bipolar spindle assembly in flies. Leading off with the sunn phenotype is somewhat misleading. This phenotype could still be mentioned, but it should come later in the paragraph after the SMC3 depletion result is described. We have changed the order to SMC3 before sunn. 
15. p.22, Auxin methods: more detail regarding the auxin experiments should be included. Was the auxin added to the molten agar before plates were poured, or was it added to plates after they were poured? What volume of auxin was added, or what concentration was the stock? What solvent was the auxin dissolved in? Were controls done to demonstrate that the phenotypes were a result of degradation of the target and not due to the solvent? Did the authors use IAA or NAA or one of the newer synthetic auxins? Degron experiments have given variable results in different labs, so it is critical to give detailed methods for these experiments. The requested details have been added to the materials and methods.
16. pp.22-23, Fluorescence Intensity Measurements: insufficient detail is given for the quantification. In particular, what was used as the reference image for background subtraction (line 16)? Was it dark noise of the camera, or a region of the image outside of the worm, or something else? This has been added to the methods. In lines 18-19, how was the diameter of CLS-2::GFP rings calculated from the area inside of an ellipse? I don't understand how this would be done, since the angle of the ring relative to the objective would vary. More detail, and ideally the equation used, needs to be given. More detail including the equation has been added. For intensity measurements done on single planes, what, if anything was done to ensure that the image plane was in the center of a chromosome or spindle and that differences in intensity were not simply due to the focal plane being analyzed? More detail has been added to the methods to explain this. For supplemental figures 1 and 2, how were the regions of the spindle or nucleoplasm and cytoplasm selected? Were they the same size and shape?
17. Fig. 4C, legend: “Ratios varied depending on the distance of the chromosomes from the objective.” Presumably, this should say the distance of the chromosomes from the coverslip, since the distance between the objective and the focal plane should not change but chromosomes deeper within the gonad will be dimmer. The legend has been changed to “coverslip”. If this is not the intended meaning, it should be stated more clearly. What is the evidence that the observed differences in this figure and others are not simply a consequence of distance to the chromosomes? Distance of a chromosome from the coverslip will be random.  The statistical tests on multiple chromosomes would not reveal a significant difference if it was due to random differences in distance from the coverslip.  Perhaps this needs to be discussed in the methods. More detail has been added to the methods. 
18. Fig. 7B, please use symbols to indicate bivalents in each subset of chromosomes described in the legend. We have now clearly labeled examples of large area and small area chromatids in the blow ups of Fig.7B.
19. Supplemental Figure 1: In panel A, “Paternal DNA” is confusing – is this from a male, or simply the sperm DNA in an unmated hermaphrodite? In panel B, “+1 embryo” is never defined. What is the importance of the mitotracker label, and what is the conclusion from the two panels on the left? It is unclear in these panelswhere the sperm DNA is, if it is shown at all.  The figure legend to now Fig. S2 has been expanded to address all of these issues.  A paragraph has also been added to the results to more fully explain this experiment.
20. It has previously been shown that the axial element proteins HTP-3, HIM-3, and HTP-1/2 and the SC central region proteins form polycomplexes in rec-8; coh-4 coh-3 mutants but associate with chromosomes in spo-11 rec-8 mutants. Thus, AE formation requires cohesin but not SCC, similar to bipolar spindle assembly. It would be interesting to know if AEs are required for bipolar spindle assembly in spo-11 rec-8 mutants. If so, it would add another layer of mechanistic insight.  HTP-1/2 were not detected on chromosomes after anaphase I by Martinez-Perez et al (2008) making it unlikely that AE are required for bipolar spindle assembly during meiosis II.  Thus adding additional triple mutant analysis with AE mutants would not necessarily clarify the mechanisms leading to bipolar spindle assembly and would definitely make the manuscript more difficult to digest for the non-expert reader.

Minor comments, wording suggestions, etc.:
1. The authors should be consistent in their use of genetic nomenclature. In the standard nomenclature for C. elegans, gene names are separated by a space when on the same chromosome and by a semicolon when on different chromosomes. In the abstract (p.2, lines 7-8) commas separate both genes on the same chromosome and genes on different chromosomes (spo-11 and rec-8 on chromosome IV, coh-4 and coh-3 on chromosome V). Elsewhere (e.g. p.7, lines 21-22) no punctuation is used in the same genotype. While the authors may choose not to follow the standard nomenclature in the text to enhance the readability, they should strive to be consistent.  We have standardized the cohesin genotypes without commas or semicolons in the results text for readability.
2. Standard nomenclature should be used in the Strain List. Mutations should be listed in order of their genomic location, from left to right on chromosome I, then chromosome II, etc. The allele designation for Karen Oegema's lab is lt (LT), not it. Thus, their transgenes begin with ltIs or ltSi (e.g. ltIs37, the widely used mCherry-tagged histone).  We have corrected the genetic nomenclature in the strain list.
3. p.5, line 22: “highly identical” is confusing. Things are identical or they are not.  We have deleted “highly identical”.
4. p.6, lines 11-12: Severson et al (2009) did show in a supplemental figure that apolar spindles assemble around the chromatids of rec-8 embryos during meiosis II.  Fig. S2 of Severson and Meyer, 2009 indeed shows a time-lapse sequence of GFP::tubulin in a rec-8 mutant.  However, the legend states: “(B). Meiotic spindles (SP) assemble during meiosis I (SP1) and meiosis II (SP2) of wild-type and rec-8 mutant zygotes.  Thus, two rounds of meiosis occur in rec-8 mutants, but polar body extrusion fails in meiosis II.”  The word apolar is not used, no spindle defect is described and a single example is shown.  Thus our statement at the end of the introduction “Although it was reported that rec-8 embryos do not extrude a second polar body, the structure of the metaphase II spindle was not described in detail.“ is accurate and fair in acknowledging previous work and setting up the reason for the current study.
5. p.7, line 19: perhaps “continuous” instead of “contiguous.”We have changed the word to “continuous”.
6. p.10, line 5: typo SMC-1::AAID::GFP. We have corrected this.
7. p.11, line 18: Is GFP::AIR-2 correct? AIR-2::GFP is used elsewhere. This has been corrected.
8. p.12, lines 6-9: this should refer to Fig. S1, not Fig. S3.  Because a new Fig. S1 has been added, all of the supplemental figure citations have been updated.
9. p.13, line 23: the -1 oocyte has been cellularized and therefore is no longer part of the syncytial gonad.  The word “syncytial” has been deleted.

10. Many citations throughout the text are formatted incorrectly. One example: “(K. McNally, Audhya, Oegema, & McNally, 2006; Srayko, O'toole, Hyman, & Müller-Reichert, 2006)” Citations have been changed to numbered “Vancouver” citations.  Numbers are currently in parentheses rather than brackets because Endnote switches to parentheses when Vancouver is selected.  This will be corrected when reviewers have finished adding references.
11. Fig. 1A: it would be helpful to add timestamps similar to those shown in stills from other timelapses.
12. Fig. 2B: the legend states that there are 24 chromatids with one chromatid visible in both slices 9 and 14. This chromatid should be somehow indicated in the figure, for example with an arrowhead.
13. Fig. 3: it is confusing to have two figure panels labeled A.
14. Fig. 6A,B: it would be helpful to add symbols to indicate spindle poles in multipolar and bipolar spindles, as well as some examples of MT fibers that are referred to.
For points #11-14, we do not think these are essential for an average reader to understand the paper.
15. Fig. 6 legend: the statement “Images in E and F have been pseudocolored for increased clarity” is confusing. Aren't all of the images in the paper pseudocolored, since they were captured with a greyscale camera? The word “psuedocolored” has been deleted from the legend.
16. Fig. 7A, it would help to have arrowheads pointing to the rings. We have added an arrowhead pointing to a representative ring.
Reviewer #2: The manuscript by McNally et.al. assesses the contribution of chromosome structure to meiotic spindle assembly, by investigating whether the bipolar structure of C. elegans chromosomes is required for spindle assembly. They therefore analyze a set of mutants with defects in sister chromatid cohesion and demonstrate that in the absence of all cohesion (spo-11, rec-8, coh-3, coh-4), spindles failed to form. However, spo-11 rec-8 mutants formed bipolar spindles in MI, presumably due to the presence of residual COH-3/4-containing cohesin complexes. In these mutants, a subset of chromosomes are able to pattern AIR-2, and chromosomes with patterned AIR-2 have a different localization of spindle factors MCAK and CLS-2. Therefore, the authors conclude that cohesin is essential for spindle assembly independent of its role in sister chromatid cohesion.

This manuscript reports interesting findings that have the potential to be of interest to the field. However, there are some major issues that need to be corrected prior to publication.

Major points:

1. The authors argue that the spo-11 rec-8 mutant has 24 chromosomes, contrary to a prior report that argued that these mutants have 12 univalents that segregate equationally in MI (Figure 2A in Severson 2009).  Fig. 2A in Severson et al., 2009 is a cartoon interpretation of polymorphism inheritance patterns.  We have changed the citation to Severson and Meyer, 2014.  Fig. 3D in Severson and Meyer, 2014 unambiguously shows the lack of sister chromatid cohesion in rec-8 spo-11 mutants by measuring the distance between GFP::lacI spots in oocytes that are heterozygous for a lacO array.  This distance is significantly greater than the same distance in rec-8 single mutants.   However, the data presented to support this conclusion (Figure 2D, 2F) is not convincing. The chromosome counting appears to have been done on metaphase-stage spindles, where the chromosomes are very close together (however, there are no details in the materials and methods describing this counting, so it is hard to tell; see point #2 below). If counting was done in metaphase, it would be difficult to definitively state that two chromosomes close together are not part of the same bi-lobed univalent (as is seen in rec-8 single mutants, and as Severson argued also occurs in rec-8 spo-11). This is especially true because it has been shown that the chromatids in rec-8 mutants are quite separated, even though they are still held together (the authors also show this in the current manuscript, Figure 4B, Metaphase I image). Therefore, the 24s counted in Figure 2F could actually be 12s, just with the individual sisters further away from each other than would be seen in WT MII univalents. If the authors want to argue that this mutant has 24 individual chromosomes, they need to demonstrate this more rigorously. They should count chromosomes in diakinesis, where the chromosomes are more spread out, We have added chromosome counts from diakinesis z-stacks to a new Fig. S1 and added more detail to the Materials and Methods. and also image additional markers to aid in determining if particular chromosomes are linked to others. I suggest imaging MEL-28, as they do in Figure 1E, and also AIR-2, as they do in Figure 4B, which may reveal chromosome connections; this imaging should be done using multiple z-stacks, to make sure they are imaging all chromosomes The distances between GFP::lacI spots reported in Fig. 3E in Severson and Meyer 2014 indicate that any connections between sister chromatids in rec-8 spo-11 mutants would be very long (majority > than 3 um).  If there are long thread-like connections between chromatids that label with MEL-28 or AIR-2, analogous to the heterochromatin connections that connect Drosophila 4^th^ chromosomes, this would indeed be interesting.  However, the data shown in Fig1, Fig. S1, and Severson and Meyer (2014) are adequate to show a lack of SCC in spo-11 rec-8 double mutants.     (unlike the single-slice images shown in many other panels, such as Figure 4D, where only a minor subset of chromosomes are shown The purpose of Fig. 4D is not to show the number of chromosomes.  The purpose of Fig. 4D is to show the uniform dim labeling by AIR-2 in the quadruple mutant and the highly variable bright/dim labeling in the double mutant.  Showing a complete z-stack would not make this clearer and Reviewer 3 point 3 wants fewer z-stacks to be shown. ). Also, the authors should provide movies stepping through the z-stacks so that the readers can see the chromosomes and count them themselves. We have added Fig. S1 with a z-stack stepping through a diakinesis oocyte. This may seem like overkill, but strong evidence is needed if the authors are attempting to dispute a prior published study. (If it turns out that this mutant does not have 24 chromosomes as the current version of the manuscript claims, the conclusions need to be substantially adjusted.) Finally, it is also important to include a better discussion of the Severson paper and provide an explanation for the discrepancies between this study and theirs so the reader can better understand this section of the manuscript. There is no discrepancy between our manuscript and Severson 2014.  The discrepancy is with the interpretation of polymorphism inheritance data in Severson 2009.  Trying to resolve an apparent discrepancy between cytological chromosome counts and PCR polymorphism inheritance patterns would be a significant distraction from the main points of this manuscript.

2. The Materials and Methods section needs more details. For example, the only imaging described is single-plane live imaging, but in some of the figures the authors show sum projections of z-stacks (e.g. Fig 2B, D). Information about the step size and acquisition of these images should be added, as well as details such as how the chromosomes were counted (e.g. Were they counted from the summed images or from going through individual stacks? How did the authors ensure they were counting two independent chromosomes vs. two that are connected? What stage was assessed?).  The requested additional details have been added to the materials and methods.

3. Similar to point #2 above, the Materials and Methods has no information about the generation of the AID strains. Have all of these strains been published before? If so, cite the relevant references in the strain list (Table S1).  All of the AID strains have been published before and references have been added to the strain list.  If not, the authors need to discuss how these strains were made (which terminus were the tags inserted into, what linkers, if any, were used, etc.), and also present a characterization of these strains. Sometimes adding a degron tag and/or GFP can alter protein function. The authors should present brood size and embryonic lethality information, to increase confidence that the tags are not affecting function and impacting the results, as well as western blots to assess the level of depletion they are achieving (especially since some of the phenotypes are not 100% and the authors note that they may not be achieving complete depletion - e.g. page 10 lines 11-12 and page 13 line 13).  Because the degron tags are on hasp-1 and smc-1, which are expressed both maternally and somatically, and because the TIR1 is expressed only in the germline, Western blots of whole worms cannot be used to accurately demonstrate the degree of depletion.  Western blots on dissected gonads are beyond our technical capabilities.  Furthermore, demonstrating the extent of depletion is essential when reporting a lack of a phenotype.  In this case, we are presenting phenotypes that are similar to those of cohesin mutants so demonstrating the extent of depletion is less critical.    

4. The authors cite Severson (2009) as showing that spo-11 rec-8 double mutants “retain COH-3/4 on pachytene chromosomes” (page 8 lines 15-18). However, I don't see that data in the cited Severson paper. If this has been shown somewhere, the proper reference should be used. We have changed the citation to Severson and Meyer, 2014.  Retention of COH-3/4 on pachytene chromosomes of rec-8 spo-11 pachytene nuclei is shown in Fig. 4A of Severson and Meyer, 2014.  However, even if it has been shown, is it known whether COH-3/4 remain on chromosomes during spindle assembly (the stage the authors are assessing in Figure 2C)? The authors need to show this (or cite the relevant study) if they want to claim that chromosomes in spo-11 rec-8 have COH-3/4 at the stage they are analyzing.  Retention of SMC-1, which is a component of both rec-8 cohesin and COH-3/4 cohesin on metaphase I chromosomes of rec-8 spo-11 mutants is shown in Fig. 3A and 3B.    
5. The MEI-1 data in Figure 2E is confusing. The authors claim that the chromosomal pixel intensity divided by the cytoplasmic intensity was not significantly increased, but the signal is so much brighter in the representative image that this is hard to believe. The raw images are 16 bit images (potentially 65,000 shades of gray).  To display images to the human eye, they must be converted to 8 bit (256 levels of gray).  The standard used for this conversion is to display the brightest pixel in the image as white and the dimmest pixel as black.  This conversion causes the chromosomes to look brighter when the spindle is dimmer.      I couldn't find any information in the Materials and Methods about how this quantification was done - adding this information is essential for the reader to evaluate this data. The method used for quantifying the pixel intensity of GFP::MEI-1 has been added to the materials and methods.  Also, even if the interpretation of the authors is correct (that the increased contrast of chromosomal MEI-1 is due to a decrease in microtubule-associated MEI-1), couldn't this result still be real (i.e. couldn't mistargeting excess MEI-1 to the chromosomes affect the ability of spindles to assemble)? Our analysis of chromosomal GFP::MEI-1 pixel intensity indicated no significant difference between bipolar metaphase I spindles and apolar metaphase II spindles in rec-8 spo-11 embryos.  It is possible that “mistargeting excess MEI-1 to chromosomes affects the ability of spindles to assemble”' however, our quantitative analysis revealed no evidence for targeting excess MEI-1 to chromosomes in metaphase II rec-8 spo-11 mutants.   The authors should look at MEI-1 localization in the quadruple mutant that is unable to assemble a bipolar spindle, and in other mutants where cohesin is disrupted and bipolar spindles cannot form (e.g. the bir-1 mutant and haspin(degron)), to see if increased MEI-1 chromosomal staining is correlated with spindle defects.  Because our data in this manuscript shows that the amount of MEI-1 on chromosomes does not correlate with bipolar spindle assembly in a rec-8 spo-11 mutant, there would still be no correlation if we found a difference in other mutants.

6. Figure 4: The authors show that in spo-11 rec-8 mutants, some chromosomes have bright AIR-2, and some only have diffuse AIR-2. However, if I understand correctly, all of these chromosomes have “non-cohesive cohesin” that is “sufficient for bipolar spindle formation” (Figure 3). Do the authors have an explanation for why some of these chromosomes are able to pattern AIR-2, while others load diffuse AIR-2 (as they show in Figure 4)? This should be explained somewhere in the text, since it is hard to follow the logic as currently written. Moreover, related to this point, I don't understand the sentence (page 13 lines 15-19) that states “the subclass of Aurora B that is recruited to chromosomes by cohesin and haspin-dependent phosphorylation of histone H3 is required for bipolar spindle assembly…”. If cohesin recruits Aurora B to chromosomes, then shouldn't all chromosomes properly load AIR-2 in the spo-11 rec-8 mutant, since the authors showed that SCC-1 is present on all chromosomes? It is possible there is a logical explanation for this that I missed, but I think the authors should explain this better to help the reader follow the logic of the experiments.  We have added a short paragraph explaining what is known about the heterogeneous loading of AIR-2 on univalents in spo-11 single mutants and suggest that bright patterned AIR-2 on only a subset of chromatids is sufficient to promote bipolar spindle assembly. 

7. Figure 4D, 4F: The localization of AIR-2 on chromosomes (diffuse vs. patterned) is not easy to see in the images presented. Also, it would be useful to know, within a given oocyte, how many of the chromosomes have diffuse vs. patterned AIR-2. In the quadruple mutant, it looks like all are diffuse, but in spo-11 rec-8, the image in 4D makes it look like some are patterned and some are not. More oocytes should be assessed for each mutant (only 5-6 oocytes are quantified and pooled in Figure 4C and 4E; more should be assessed) and in addition to the pooled data presented, the authors should report the numbers for each oocyte with appropriate statistics, so it is clear how much this number varies from oocyte to oocyte.
We have added new Fig. S2B which shows the number of bright vs dim AIR-2::GFP chromatids within individual nuclei and magnified images of bright vs dim AIR-2::GFP in Fig. S2A.  We have also added additional information in the materials and methods.  Fluorescence intensity was only measured objectively in focal planes with similar cytoplasmic or nucleoplasmic intensity to compensate for the reduced intensity of chromosomes further from the coverslip.  This is why n is low for objective intensity measurements.  N is much higher in Fig. S2B where subjective counting of bright vs dim in the same focal plane allowed scoring all of the chromosomes within a nucleus.   
8. In Figure 5, the authors characterize a bir-1 mutant that, based on work in other organisms, should not properly localize to chromosomes (page 12, lines 15-18). However, this has not been shown in C. elegans. The authors should stain for BIR-1 in the mutant, to confirm that there is no chromosomal staining (to make sure that the interpretation of this figure is correct).  It is well established that auroraB is in a complex with INCENP and survivin (BIR-1).  Thus the reduced localization of AIR-2 in the bir-1 mutant shown in Figure 5C and 5E is a proxy for showing reduced BIR-1 localization.  The presence of AIR-2 on anaphase microtubules in 5B indicates that BIR-1 protein is still present in the bir-1 double point mutant because AIR-2 would not show any localization in the absence of BIR-1.

9. Figure 6C: In the results section (page 14 line 7-9) it is stated that microtubule bundles in the bir-1 mutant do not coalesce, but the movie shown only goes to 1:30, when control spindles have not coalesced either, so this is not convincing. The figure has been changed to show failed spindle assembly over a longer time period for the cohesin quad mutant.

10. The different patterns of MCAK on univalents in Figure 7B and 7D are hard to distinguish - even the zoomed images just look like dots or blobs, making it hard to tell what MCAK actually looks like. Higher resolution images are necessary for the reader to understand this localization pattern. We are not able to determine a specific localization pattern for AIR-2, KLP-7, or CLS-2 on bipolar spindle competent single chromatids.  This is not just a matter of resolution.  It is also hampered by movement and random orientations in live imaging.  There is also a very real possibility that it is not a uniform pattern.  Published work on rec-8 univalents indicate they have poorly defined, possibly transient recombination intermediates that may not have a uniform pattern.  This is why we measured intensity (for AIR-2) area (for KLP-7) and diameter (for CLS-2).  We could demonstrate a statistically significant difference with these measurements.
Also, similar to point #7 about Figure 4, the authors should increase the number of oocytes analyzed and also report, within a given oocyte, how many chromosomes have patterned vs. diffuse MCAK.  
n has been increased from 6/60 and 6/33 to 8/75 and 7/43.  As described for other points, only some focal planes of some z-stacks are suitable for quantification.  The number of chromatids within a nucleus with bright AIR-2 is now in new Fig. S2.  The correlation between AIR-2 and KLP-7 in Fig. 7E,along with Fig.S2B allows the reader to gauge the variability in the number of chromatids with patterned KLP-7.  The only conclusion that we draw from this variability in the results is that not all chromatids have to have bright patterned AIR-2 to drive spindle assembly.
11. The CLS-2 data in Figure 8A is not convincing. Specifically, page 16 lines 10-12 state “cohesin-dependent AIR-2 excludes CLS-2 from the midbivalent ring…”. However, the data presented in Figure 8A does not show that CLS-2 localizes to the ring in the bir-1 mutant - there is only a small amount of CLS-2 on the sides of the bivalent, but this does not extend all the way across, as would be expected for a ring protein. It has been shown that when you deplete AIR-2 to prevent formation of the RC, the midbivalent break between the two homologs is gone (Monen 2007 and Divekar 2021; this is also apparent in the mCH:his images in Figure 8A). Under these conditions kinetochore proteins change from two distinct cups, to one continuous pattern surrounding the bivalent. This is what I think the authors are seeing in Figure 8A. CLS-2 is not loading onto the ring (because there is no ring complex if the CPC is not present); instead, the kinetochore staining just spreads further into this middle region because it coats the entire bivalent. If the authors really want to demonstrate that CLS-2 has RC localization, they would have to show more convincing images, with staining all across the midbivalent region, and also co-localization with another RC component.  We have revised this results section to point out that our control images are consistent with Pelisch 2019.  We have added additional focal planes to Fig. 8A and added supplemental videos of z-stacks to clearly show that CLS-2 invades the midbivalent region in the bir-1 mutant.  We have changed the words “midbivalent ring” to “midbivalent region” to satisfy the reviewers assertion that there can be no ring if there is no CPC.

12. Related to point #11, the localization of CLS-2 to the midbivalent has not been replicated by others since the Dumont 2010 study, so it is unlikely that CLS-2 is an RC protein (see Pelisch et.al. 2019, Figure 6A, and the control images for Figure 8A in the current manuscript). However, in the discussion CLS-2 is stated to be a RC protein (page 19 lines 19-20) - this should be corrected and Pelisch should be cited. We have revised this section of the discussion.

13. The authors show that there is a positive correlation between the presence of AIR-2 and the recruitment of CLS-2 into larger spheres (Figure 8A). However, is it possible the “larger spheres” just represent a difference in chromosome organization in the presence and absence of AIR-2, rather than a specific effect on patterning CLS-2? Maybe the chromosomes are larger in the presence of AIR-2? The authors should measure chromosome diameter in the presence and absence of AIR-2, to rule out this possibility. Moreover, they should provide a possible explanation for the larger diameter of CLS-2 - this reviewer was left wondering what this pattern meant…what is the significance of a “larger diameter”? To address the question of whether chromosomes are just larger when they have air-2 loaded, we have added analysis of the diameters of mCherry::h2b to Fig.8D and added this possibility to the concluding sentence of the corresponding results paragraph.  
Other points:
- It is hard to keep track of chromosome configurations in the various chromosome structure mutants, especially for people outside the field. I suggest adding more diagrams, similar to those shown in Figure 1A comparing WT and rec-8, but for the other chromosome structure mutants as well (spo-11, spo-11 rec-8, and the quadruple mutant). However, the authors should be careful making these diagrams because in Figure 1A there are thin white lines down the center of some of the single chromatids (e.g. dividing the single chromatids at the end of Anaphase II in the WT and dividing the single chromatids in the rec-8 mutant) – when I first looked at them, I thought that these were put there intentionally to distinguish sister chromatids from each other; it took me awhile to figure out that they were probably mistakes. I suggest remaking this diagram to remove these thin lines. The white lines have been removed from the cartoon chromosomes in Fig. 1B.

- Abstract line 13-15: “which regulated the localization of the spindle assembly factors CLASP-2 and kinesin-13 to mediate bipolar spindle assembly.” This makes it sound like these are the key factors that mediate spindle assembly in your mutants, which has not been demonstrated. Moreover, both CLASP-2 and kinesin-13 target to chromosomes lacking AIR-2 (the pattern or circumference is just different), so I am not entirely convinced that the changes in localization observed would affect spindle assembly. We have changed the abstract to more accurately describe the results. 
- Figure 1D: It would be helpful for the reader to show ASPM-1 in a control embryo, for comparison.Control ASPM-1 images have been added to the figure.
- Page 10 lines 18-20. This sentence talks about PDS5, but there is no reference, and it is not clear what organism is being discussed - has this been shown in C. elegans? If not, it may be best to remove discussion of PDS5 because it might confuse the reader. There is a similar reference to PDS5 on page 13 lines 14-15.  Direct interaction between PDS5 and haspin was shown in the Yamagishi 2010 reference which was included on page 10-11 with 2 other references at the end of the second sentence describing the entire pathway linking cohesin to the CPC.  We have moved the Yamagishi reference closer to the word PDS5.  The Yamagishi reference is now also included directly after the word PDS5 on page 13. We cannot remove reference to PDS5 because it is a well established link between cohesin and haspin and is essential for the reader to understand the logic of the paper.

- Figure 3C: The authors image LIN-5 in Figure 3C and 5D (presumably using it as a pole marker) but they provide no information in the results section about what LIN-5 is or why they are imaging it; this information should be added to make the manuscript more accessible to non-experts.  We have added the sentence: “For this experiment we monitored endogenously tagged GFP::LIN-5 as a spindle pole marker instead of GFP::ASPM-1 because the  aspm-1 gene is linked to smc-1/him-1.”
- Figure 4B: the authors state in the text that AIR-2 is bright on microtubules in Anaphase II (page 11 line 12) but the image shown appears to be end-on and therefore gives the impression that AIR-2 is on chromosomes not microtubules. This localization should be shown more convincingly. Figure 5B has the same problem - AIR-2 appears to overlap with chromosomes in Anaphase II but the text says that AIR-2 is on microtubules.  The images do not get any better.  The localization of AIR-2 clearly broadens to fill spaces between chromosomes during anaphase.  We have changed the description in the results and figure legends for Figs 4 and 5 stating that this broadening beyond the chromosomes is consistent with transfer to microtubules.

- Fig S1: I was confused by the discussion of this figure in the results section. Page 12 lines 7-8 states that “sperm-derived paternal DNA within meiotic embryos recruited maternal GFP::AIR-2 but lacked detectable cohesin and did not promote spindle assembly”. However, the authors should explain the experiments presented in Figure S1 in more detail if they want readers to understand this point (the mating experiments are not described except in the Figure S1 legend, and this is not enough information to make this experiment accessible to a broad audience). We have added a paragraph to the results section and expanded the figure legend to Fig now S3.
- The section on spindle assembly (starting on page 13 line 21) does not reference prior studies describing the wild type spindle assembly pathway - for example, page 14 lines 3-4 notes that microtubule bundles normally arise within the germinal vesicle, but prior studies describing this are not referenced (Wolff et.al. and Gigant et.al. 2017). We have added the appropriate references.
- The authors note that MCAK targeting to the midbivalent is dependent on bir-1 (page 15, lines 12-14). Since it has been shown that MCAK targeting to this region requires AIR-2 (Divekar, et.al. 2021), this previous (similar) result should be cited.  The Divekar citation has been inserted here.
- Figure 7: the images of MCAK and AIR-2 colocalization are small and hard to interpret. Does the MCAK pattern match the AIR-2 pattern? Higher resolution images are needed to better understand this result. We are not able to determine a specific localization pattern for AIR-2, KLP-7, or CLS-2 on bipolar spindle competent single chromatids.  This is not just a matter of resolution.  It is also hampered by movement between image captures and random orientations in live imaging.  This is why we measured intensity (for AIR-2) area (for KLP-7) and diameter (for CLS-2).  We could demonstrate a statistically significant difference with these measurements.
- Some of the reported Ns are low. For example, one of the conditions in Figure 3B and 8C only has 3 embryos. A minimum of 5 should be reported (though for some experiments, where there is more variability, this should be even higher; see major points #7 and #10).
We have increased n for the low N values.  
Typos:
- Page 6 line 4: “individual” is misspelled This has been corrected.
- Page 10, line 5: “AAID” should be “AID” This has been corrected.
- Page 12 line 9: “Fig S3” should be “Fig S1” This has been corrected.
- Page 15 line 17: “Fig. 6B, C” should be “Fig. 7B, C” This has been corrected.
- Page 15 line 20: “Fig. 6D, E” should be “Fig. 7D, E”. This has been corrected.
- Page 16 line 7: “Fig. 7B, C” should be “Fig. 8B, C”. This has been corrected.
- Page 16 line 7: “Fig. 7D, E” should be “Fig. 8D, E”. This has been corrected.
- Sometimes you use “Ran” and sometimes “ran”. Make this consistent.  All Ran's have been capitalized.
- Figure 2E: label the top row “merge” This has been added.
Reviewer #3: The results in this manuscript reveal a role for cohesins in promoting the localization of the chromosome passenger complex (CPC) to the chromosomes and spindle assembly. A key result is that when cohesins are absent, an amorphous cloud of microtubules is observed instead of a bipolar spindle. In MI, this requires depleting both rec-8 and coh-3/4. In MII, because coh-3/4 is normally absent, depletion of only rec-8 leads to a loss of spindle formation. This paper includes a nice result showing auxin-induced depletion of SMC-1 leads to a similar phenotype. In addition, there is strong evidence linking cohesins to CPC recruitment (eg. Fig 4). The main conclusions of this paper are supported by the data. A significant problem, however, is redundancy between figures, lack of raionale for certain genotypes, and a couple experiments that do not show exactly what the authors claim, or the implications are ambiguous. These issues and others listed below should be addressed prior to publication. In short, this paper could be improved with a more concise presentation.

1) Why is the spo-11 mutation in most of the cohesin genotypes. For example, why is there no rec-8; coh-3 coh-4 mutant. It seems that the presence of a spo-11 mutation should not be required to observe the defects in spindle assembly. Besides the presence or absence of the spo-11 mutant may not effect spindly assembly, the results in Fig 2C seem to duplicate results in Fig 1. What does Fig 4F (spo-11 rec-8) show that is not shown in Fig 4B (rec-8) (this results is also not measured or quantified and it should). The key comparison in this paper is metaphase I spo-11 rec-8 (no SCC but yes cohesin) to metaphase I spo-11 rec-8 coh-3 coh-4 (no SCC and no cohesin).  This comparison is what demonstrates that cohesin's bipolar spindle assembly function is independent of its role in SCC.  
2) Figure 1D or E lacks controls. For example, the localization of ASPM-1 is not shown in controls. We have added a control for the ASPM-1 localization.  There is also no rationale given for examining MEL-28 localization. What does this result show? The question posed in the introduction is whether the bipolar structure of bivalents and univalent is required for bipolar spindle assembly.  The MEL-28 figure shows that bipolar-spindle-incompetent separated sister chromatids in a rec-8 mutant lack bipolar symmetry.  We have modified the sentence in the results to clarify this connection.  The results in Fig 2E are more relevant and could be move to Fig 1 instead (see below). The conclusion is the same as with ASPM-1, and 2E has a control.
3) Are all the image slices needed in some of the figures, like in Fig 2? I understand this is to see how many chromatids there are, but the key result here is the microtubules, not the number of chromatids. The extra slices could be in a supp figure.  Reviewer 2 major point #1 was not convinced that we are showing enough z-planes.  We have added additional Z planes to new Fig. S1.  The key result is the microtubule configuration when there is no sister chromatid cohesion (SCC) so these chromosome counts are critical to the main point of the paper.
4) Pg 10, line 2: is there evidence that coh-3/4 is a target of separase? If so, this should be cited. In contrast, there are meiotic cohesins that do not appear to be cohesin targets.  We have removed “by separase” in response to a similar question from another reviewer.
5) Some stats in Fig 4C are borderline (p=0.07) although the most important comparison is significant. The sample size is rather small and the authors should try and improve this. There is no argument in the text based on the p=.07.  The argument in the text is based on the comparison of p=.75 to p=.0001.  The intensity of AIR-2 decreases between MI and MII in rec-8 but not in controls.  In Fig 4D, why is MI not shown?  The oocytes shown are just before initiation of spindle assembly and metaphase I would be after completion of spindle assembly.  Both are informative but we do not see a compelling reason to change this particular figure.
6) Figure 5: The differences in Fig 5B with AIR-2 localization are a little hard to see. This is a case where side by side images with wild-type would be warranted and quantitation of the AIR-2 localization pattern done if possible. We have modified the Fig. 5 legend to specify where control images are in earlier figures.  The differences in Fig 5C are measured but seem less impressive and are also prior to meiosis, when different factors could regulate CPC localization.  The intensity of AIR-2 on chromosomes is relevant to spindle assembly in -1 oocytes (data shown in 5E) because this is just before (0-23 min before) initiation of spindle assembly.
7) Figure 5D: be clear in the legend why there are two images here. I believe one shows apolar and the other shows multipolar. We have added this clarification to the legend.
8) Figure 6 and text on pg 14: This seems to mostly repeat results in previous Figures. Improve the description to make it clear what is the new finding in this figure, or remove, or move to supp figures. We have expanded the text introduction to this section with references requested by another reviewer.
9) Pg 15, line: Should this be Fig 7B,C? Same for line 20. This has been corrected.
10) Figure 7A is important. In contrast, Figure 7B shows cohesin is required for Klp-7 recruitment, not Air-2. For the non-worm person, what is the difference between Figure 7B and D. Both are MI, but one is an embryo. And I really don't know what the graph in Fig 7E shows. There is no comparison to a control and it is only showing a correlation between Air-2 and Klp-7. But not a relationship to rec-8. I think 7D and E should be deleted.  The most interesting genotype in the manuscript is spo-11 rec-8 because single chromatids can drive bipolar spindle assembly.  In this double mutant, AIR-2 loading is heterogeneous with some bright and some dim chromosomes.  We have added an extra section to the results to highlight this heterogeneity. 7B is KLP-7/histone H2B labeled. Fig. 7D is KLP-7/AIR-2 labeled.  These figures show that the subset of chromatids with bright AIR-2 have an altered pattern of KLP-7.
11) Figure 8B has the same issue as Figure 7B, and 8D,E have the same issues as 7D,E.  Same explanation.
12) Pg 16-17: I don't see how these results exclude a role for the Ran pathway. The fact that the listed SAF proteins are cytoplasmic does not inform on the ran pathway. See below regarding how CPC could derepress cytoplasmic factors.  In the canonical Ran pathway (referenced to an Isabel Vernos review), SAFs are inhibited by importin binding to an NLS on the SAF.  The vast majority of defined ran targets thus have an NLS and are nuclear during interphase.  In the Vernos review, she points out that the C. elegans homolog of TPX2 has no NLS.  TPX2 is the most-studied ran target in Xenopus (where all the seminal work on the ran pathway was done).  We have clarified this logic and softened the conclusion.
13) Pg 17, line 20: there is no metaphase III. Use a different term. We have changed the wording.
14) Pg 18: The authors should cite work from Ohkura in Drosophila (Rome 2019, Beaven 2017) showing strong evidence for suppression of cytoplasmic SAFs that is alleviated by CPC activity. We have incorporated Beaven 2017 into the revised discussion. There is no Rome 2019 and Rome 2018 does not directly relate to the CPC or cohesin so we have not reviewed this paper in the discussion. The Xenopus data showing CPC inactivates MCAK is really a isolated result and these experiments did not rule out activation of SAFs. The genetic suppression of CPC loss by kinesin-13 depletion has been reported in both Xenopus and Drosophila.  These studies are both referenced in the revised discussion. In contrast, the CPC is required for kinetochore assembly is multiple systems.  Kinetochores are not required for spindle assembly during C. elegans meiosis and Air-2 is not required for kinetochore assembly during C. elegans meiosis so we are not including this in our discussion.
15) Pg 19 top: Published evidence suggests the ran pathway does not drive spindle assembly in Drosophila oocytes. The authors should discuss how there are several mechanisms to recruit the CPC to the chromosomes in other organisms. The strong role for cohesin described here may be due to the structure of C. elegans meiotic chromosomes. In Drosophila a different chromatin feature rather than cohesins recruits the CPC to the chromosomes (Wang 2021). It is striking, however, that in both C. elegans and Drosophila, recruitment of the CPC is critical for spindle assembly. Mouse also do not require Haspin for spindle assembly. We have tried to present a more comprehensive comparison between what is known in mouse, Drosophila and C. elegans.

Have all data underlying the figures and results presented in the manuscript been provided?
Large-scale datasets should be made available via a public repository as described in the PLOS Genetics data availability policy, and numerical data that underlies graphs or summary statistics should be provided in spreadsheet form as supporting information.
Reviewer #1: Yes
Reviewer #2: Yes
Reviewer #3: Yes

PLOS authors have the option to publish the peer review history of their article (what does this mean?). If published, this will include your full peer review and any attached files.


If you choose “no”, your identity will remain anonymous but your review may still be made public.


Do you want your identity to be public for this peer review? For information about this choice, including consent withdrawal, please see our Privacy Policy.
Reviewer #1: No
Reviewer #2: No
Reviewer #3: No
 


In compliance with data protection regulations, you may request that we remove your personal registration details at any time. (Remove my information/details). Please contact the publication office if you have any questions.
