# Supplementary material for: Cohesin is required for meiotic spindle assembly independent of its role in cohesion in C. elegans
Source: PLoS Genet. 2022 Oct 24;18(10):e1010136. doi: 10.1371/journal.pgen.1010136 (PMC9632809; doi:10.1371/journal.pgen.1010136)
Supplement: Attachment — Submitted filename: response to reviewers.docx [file pgen.1010136.s019.docx]

Reviewer #1: The resubmitted manuscript from the McNally lab has undergone significant revisions, including textual changes in response to reviewer’s comments that greatly improve the readability of the manuscript and the clarity of certain key points; new analyses and/or figures of prior datasets that address some of the reviewer concerns; and an increase in numbers of oocytes analyzed in certain cases where the number was initially quite low. Consequently, the manuscript is much improved. Unfortunately, many important reviewer comments have been inadequately addressed, and some textual changes raise new concerns.

My page numbers below refer to the position in the 128 page long combined PDF, not the page numbers that appear "printed" on each page, which are inconsistent between the section showing the revised draft without changes highlighted and the version with changes highlighted

Major Comments:

1. P. 109, top: “Bipolar spindle assembly 1 occurs in knl-1(AID) knl-3(AID) tir1 worms (Danlasky et al., 2020) and in worms with no degron tag treated with auxin using the same protocol. Thus the spindle assembly defect observed for hasp-1(AID) cannot be due only to non-specifc effects of auxin or the 1% ethanol solvent.” This text was added to give greater detail regarding controls that were done for the auxin degron experiments. It essentially says that experiments done by other people using other lines did not reveal any issues. This does give some level of confidence that the observed results are not a result simply of growth on plates with auxin or the carrier. However, a proper control would be done by the same person, in parallel with the experimental group, using the same batch of media, and would show the phenotype of worms expressing the SAME tagged protein and TIR1 on “normal” plates and worms with TIR1 but without the tag on the same batch of auxin plates. Because proper controls were not done, the word “cannot” is untrue and must be replaced with something else. Perhaps, “Thus, the spindle assembly defect observed for hasp-1(AID) likely does not result from non-specific effects…”

We have added no auxin controls to the figure for both degrons, describe additional controls in the materials and methods, and have changed the wording to that suggested by the reviewer.

2. Also regarding controls, text was added to the legend for Figure 5 (p. 123) to specify where “control images” are in earlier figures. Unless the experiments were done at the same time, and with only one variable changed from the experimental group, I do not believe the earlier images can accurately be called control images. Moreover, the legend states that “Control for 5D is in Fig. 3C.” Fig. 5D shows mCH::H2B and eGFP::LIN-5, while Fig. 3C shows mKate::TBB-2; SMC-1::AID::GFP; eGFP::LIN-5 in the presence and absence of auxin. These are completely different strains, although I assume the lin-5 transgene is the same. 3C most certainly should not be called a control for 5D. Although it is clunky, the authors must describe the comparisons accurately. Perhaps “eGFP:LIN-5 localization in a different strain, in which a different protein is tagged with AID, is shown in Fig. 3C.” Similar wording is needed to replace “control” for the other parts of this figure to avoid the risk of misleading the reader. A better alternative would be to do the proper controls.
Minus auxin controls for the hasp-1 degron have been added to Fig. 4D, 6C and 6D.

1A is in fact a proper control for 5A because the only difference between these 2 strains is the double point mutation in bir-1. 4A is in fact a proper control for 5B since the only difference between these strains is the double point mutation in bir-1. It is completely impossible for us to film controls “at the same time” as experimental conditions because acquiring one high quality time-lapse sequence frequently requires 8 hours of filming. We have added the following sentence to the materials and methods. “Control and experimental time-lapse data sets always included sequences acquired on multiple different days”.
3. Regarding my comment 11 in the first review: The suggestion that the dependence of bipolar spindle assembly on cohesin might prevent the formation of a metaphase III spindle is still quite confusing. In metaphase I and II, cohesin is not required for spindle MT assembly, just for bipolarity. There is never a third round of spindle assembly/MT assembly between anaphase II and pronuclear migration, even in cohesin triple mutants. Why would a requirement for cohesin in promoting bipolarity matter when a third round of MT assembly doesn’t occur? Adding the word "bipolar" or changing the wording from “meiosis III” to something different doesn’t increase the plausibility of this model.
We have deleted this from the discussion.
4. Responses to several reviewer comments stated that experiments could not be done because of technical limitations, when the experiments could easily be done if immunofluorescence were used instead of live imaging. These include:

a. Reviewer 2 comment 3 suggested evaluating the level of depletion by AID using a Western blot. The authors response is correct that Western blotting might not give a clean result because TIR1 is expressed only in the germline while the AID-tagged protein is expressed in both soma and germline. However, level of knockdown could be addressed by fixing worms and staining with antibodies to SMC-1 and HASP-1 (SMC-1 antibodies definitely exist; if antibodies to HASP-1 don’t yet exist, there are great antibodies to the Histone H3-PhosT3 mark that HASP-1 makes and to the AID tag used in this study).

We have added a new supplemental figure S2 documenting depletion of SMC-1::AID::GFP (in oocyte nuclei because the GFP::LIN-5 marker in the strain is excluded from nuclei) and reduction of phosphor H3T3 staining after HASP-1 depletion. We also have added hatch rate data in new Table S1 and compare with the complete sterility of null mutants as evidence of partial depletion.

b. Reviewer 2 comment 10: patterns of MCAK on univalents are hard to distinguish. It is undoubtedly difficult to get good images of this via live imaging because as the authors state in their response, chromosomes and embryos are moving. However, there are very good antibodies to MCAK, and movement would not be a problem in fixed and stained gonads, so the reason given for not addressing the issue is not convincing. c. Same for Reviewer 2 comment on Fig. 7 in “Other points.” The concern could easily be addressed by immunofluorescence.

Old Fig. 7E (new Fig. 8E) has been replaced with an Airyscan super resolution image of a fixed embryo stained with anti-KLP-7 and with anti-GFP to enhance GFP::AIR-2.

d. Reviewer 1 point 6: do chromatids with intense AIR-2 signal also have high levels of COH-3/4. Although this could not be addressed in live imaging because COH-3::mCherry was too dim, there are very good antibodies for both AIR-2 and COH-3/4, so the reason for not addressing this is not convincing.
AIR-2 and COH-3/4 co-localization by fixed immunofluorescence is now shown in a new supplemental Fig. S4.

Minor Comments:

1. Figure 1B: I agree with reviewer 2 that showing a similar cartoon for spo-11 rec-8 and for spo-11 rec-8 coh-4 coh-3 would be quite helpful for unfamiliar readers to understand the model for these mutants. Also in this figure, the legend does not state what is meant by the blue and green lines. What are “blue cohesin” and “green cohesin”?

A cartoon has been added to new figure 2. We have changed the colors of cohesin and added explanation to the legend of figure 1.

2. I agree with the authors that it is important to mention PDS5, since it is the connection between haspin and cohesin. However, I also agree with reviewer 2 that it is important to state in which organisms/cell types the role of PDS5 has been shown and whether it has only been shown in mitosis or also in meiosis. “in mitotically proliferating human cells…”

To address this concern, we have added the following text to the results: “During mitosis in cultured human cells and fission yeast (Goto-2017), cohesin-associated PDS5 recruits haspin kinase to chromosomes (31) and the recruited haspin phosphorylates histone H3 threonine 3. Although PDS5 has important functions during meiotic prophase in several species (Joo 2022; Pradillo 2015; Ding 2016; Kusch 2015), a role in recruiting haspin during meiosis has not been reported to our knowledge.”

Reviewer #2: This revised manuscript is much improved, and the major issues have been fixed. Notable improvements include increasing the number of n’s in key figures, better explanations of the quantification schemes, and quantification of the number of chromosomes with patterned Aurora B (Figure S2). I have a few remaining suggestions to improve the manuscript prior to publication, which are all minor compared to the original review and do not require any additional experiments.

Specific points:

- In Major point #5 from my original review, I had noted that it was confusing that the MEI-1 signal looked bright on chromosomes, yet the quantification suggested that it was not increased. I appreciate the addition of details to the materials and methods about how this quantification was done, as well as the explanation provided in the response document explaining the issue that arises when 16 bit images are converted to 8 bit – these clarifications made the data more convincing to me. However, I predict that some future readers will be confused by the images presented, as I was initially, without the provided explanation for how the chromosomes get brighter during the conversion process. Therefore, I think it would be worthwhile to provide the explanation (given in the response document) somewhere in the manuscript as well (materials and methods or figure legend).

We have added the following to the Methods: “This method was also used to determine the intensity of GFP::MEI-1 on chromosomes reported in the text of the results corresponding to Fig. 2E. MEI-1 looks brighter on the chromosomes in the *spo-11 rec-8* metaphase II image because the microtubule-associated MEI-1 is dimmer and the original 16 bit image (65,000 grey levels) has been scaled to display the brightest pixel as 256 in the 8 bit (256 grey levels) figure panel. The chromosomes are not actually brighter as explained in the Results.

- In Figure 3, two panels are labeled “A”. Reviewer 1 commented on it, but this was not fixed. (In my readthrough of the revision, I noticed this too, and I agree with reviewer 1 that it is confusing.)

This has been fixed in the figure which is now Fig. 4.

- Page 10, lines 14-16: the text states that “SMC-1:AID:GFP was found on control metaphase I and metaphase II chromosomes and metaphase I chromosomes of spo-11 rec-8 mutants…” However, when looking at the graph shown in Figure 3B, it looks like a fair number of measured chromosomes in the spo-11 rec-8 mutant in MI fall close to a ratio of 1 (which would indicate no enrichment on chromosomes). I am very convinced that many of the chromosomes have SMC-1, but the raw data suggest that some chromosomes do not. If the authors think this is a valid point (i.e. they agree that some chromosomes may not be enriched for SMC-1 in this condition), then they should add the word “some” or “most” chromosomes when describing their data, and then comment on why some chromosomes may not have SMC-1 (since this is not obvious to this reviewer).

We have added the following text to the results: “SMC-1::AID::GFP was found on control metaphase I and metaphase II chromosomes and on most metaphase I chromosomes of *spo-11 rec-8* mutants but was absent from the metaphase II chromatids of *spo-11 rec-8* mutants (Fig. 3A, 3B, 3C). The absence of SMC-1 from a subset of metaphase I *spo-11 rec-8* chromatids may be due to WAPL-1-dependent and WAPL-1-independent pre-anaphase removal pathways (Crawley et al., 2016).

But if the authors think that all chromosomes have SMC-1 and there is a different explanation for the chromosomes with a ratio close to 1, then they should provide this explanation in the materials and methods, when describing their quantification scheme. We chose the first option and the explanation is above.

- Page 10, line 20: there is a reference to smc-1/him-1, but non-worm people will not know what him-1 is. Either explain this or remove the “/him-1”.

We have deleted “him-1”

- There is inconsistency in how “Aurora B” is written. In the discussion in particular, “auroraB” is used frequently. Unless there is a specific reason for this, I suggest “Aurora B” should be used throughout the manuscript.

We have changed the name to “Aurora B” throughout the manuscript.

- I had suggested that the authors provide more diagrams, similar to the ones in Figure 1A, showing chromosome structure in the other mutants (spo-11 rec-8, and the quadruple mutant). Although the authors did not take my suggestion, I still think that this would help readers who are not C. elegans meiosis experts keep track of chromosome organization in the various conditions, helping them understand the results. Therefore, I would like to again gently encourage the authors to consider this. However, I do not consider this essential for acceptance, so I leave it up to them.

A cartoon has been added to new figure 2.

- I still think that it would be helpful if the authors state in the text what organism the PDS5 statements are referring to (in the Yamagishi 2010 reference). Without stating this, readers might think that the sentences are referring to C. elegans PDS5.
To address this concern, we have added the following text to the results: “During mitosis in cultured human cells (31) and fission yeast (Goto-2017), cohesin-associated PDS5 recruits haspin kinase to chromosomes (31) and the recruited haspin phosphorylates histone H3 threonine 3. Although PDS5 has important functions during meiotic prophase in several species (Joo 2022; Pradillo 2015; Ding 2016; Kusch 2015), a role in recruiting haspin during meiosis has not been reported to our knowledge.”

Typos:
- Pg. 4 line 19: Ran should be capitalized

Ran has been capitalized.
- Pg. 16 line 7/8: the word “or” is duplicated

One or has been deleted.
- Pg. 20 line 10: “non-phosphorylatable” is misspelled

This has been corrected.
- Pg. 24 line 17: “non-specific” is misspelled

This has been corrected.
- Pg. 37 line 23: “dependingon” should be fixed

This has been corrected.

Reviewer #3: This is a very interesting paper demonstrating a link between meiotic cohesins, CPC recruitment, and spindle assembly. The authors have made many changes in response to the previous review, but there are still some issues they did not adequately address.

1. The author response that the key comparison is between spo-11 rec-8 and spo-11 rec-8 coh-3 coh-4 does not address the original question of why spo-11 is in there. What if spo-11 influences the phenotype? Just one image of rec-8 coh-3 coh-4 would suffice. It may also help the reader to know why the spo-11 mutation was included. For example, to easily see the effects of the cohesin mutants.
We have added a new Figure 2 with analysis of a *rec-8 coh-4 coh-3* triple mutant and we have added corresponding text to the results.
5. Minor point, the reason to show MI images is that the regulation of CPC is known to be different between prophase and metaphase. Additional factors can recruit the CPC in metaphase. And clearly MI is the more relevant time point (see also previous comment #6 and Fig 5C). Agreed the results could easily be the same. Just a thought. From the reader’s perspective, they may wonder, why 4D is one stage while 4F is another

This is explained in the text of the results. The diakinesis images show what AIR-2 looks like 1-23 min before initiation of spindle assembly. The metaphase I images (bipolar spindle competent) for rec-8 and spo-11 rec-8 are needed to compare with metaphase II (bipolar spindle incompetent).

(or why 5C is at this stage).

The spindle stages in 5B are important to show AIR-2 transferring to microtubules (or a broad pattern consistent with microtubules) whereas the diakinesis stage shown in 5C is relevant because this is 1-23 min before initiation of spindle assembly.

The authors do not do a good job explaining this.

We have done the best we can without making the text excessively long.

Can the authors speculate on why in 4C the chromosomal Air-2 is higher in rec-8 MI relative to controls?

We have added the following to the figure legend: “The higher than control intensities in *rec-8* MI might be due to the previously reported (23) expanded ring structure between chromatids which might involve unresolved synaptonemal complex intermediates (25, 27).” The use of the word “might” indicates that we are speculating which is what the reviewer asked for.

Also on Figure 4, can they comment on why the spo-11 rec-8 genotype in 4E show a mix of Air2 intensity, some like the control, some like spoc-11 rec-8 coh3/4.

We include the following to address this issue: “The reason for the heterogeneity of AIR-2 loading on separated sister chromatids of *spo-11 rec-8* mutants is not known, although it correlates with the heterogeneity of residual COH-3/4 cohesin (S4 Fig). The heterogeneity of AIR-2 loading on the 12 univalents of a *spo-11* single mutant correlates with heterogeneity in retention of LAB-1 and protein phosphatase 1, which remove haspin-dependent histone H3 T3 phosphorylation (40, 44, 45).”

10) Fig 7A confirms previous results the authors cite. The results in B and C are consistent with these observations but indirect. Therefore, the statement in lines 4-6 pg. 17 applies to Fig 7A only. 7B does not test Air-2 and 7C is only a correlation.

We have changed the subheading in the Results text to: “**Cohesin-dependent Aurora B kinase correlates with altered localization of spindle assembly factors on meiotic chromosomes”.**

We have changed the concluding sentence of the paragraph to: ” This result indicated that a subclass of bright patterned AIR-2 that is cohesin-dependent, and that correlates with bipolar spindle assembly, also correlates with a subclass of KLP-7 on chromosomes.”

However, the author response did not really answer the original question. Fig 7C needs a control. What if the KLP-7 measurement looks the same in wild-type?

A wild-type control has been added to new Fig. 8D. Wild type will have bivalents (4 chromatids) at MI and univalent (2 chromatids) at MII whereas the spo-11 rec-8 mutant has separated sister chromatids (1 chromatid) at both MI and MII. Therefore the area of bivalents was divided by 4 and the area of univalents was divided by 2. This is explained in the revised legend.

Similar comment for 7E. And shouldn’t the data in 7B-C also be extractable from 7D? Its also not clear why Klp-7 was not observed in rec-8 coh-3 coh-4, which should have the most dramatic effect.
8E and &F only show spo-11 rec-8 because this is the only genotype with a mixture of bright and dim AIR-2. If all chromosomes have bright AIR-2 (eg wild type) or all dim AIR-2 (triple or quad mutant), a correlation does not make sense.

12) The authors present no evidence on the Ran pathway, even though I agree with the conclusions. For example, being cytoplasmic/ outside the nucleus does not show something is not regulated by the Ran pathway. I think these results are interesting but not because of Ran. Its because these SAFs must be inactive while in the cytoplasm, to avoid bundling of microtubules prior to spindle assembly. And it’s not NEB that triggers spindle assembly, it is exposure of these SAFs to the chromosomes (ands their cohesin recruited CPC) that activates them.

We have rewritten this section.
