# Supplementary material for: Cohesin is required for meiotic spindle assembly independent of its role in cohesion in C. elegans
Source: PLoS Genet. 2022 Oct 24;18(10):e1010136. doi: 10.1371/journal.pgen.1010136 (PMC9632809; doi:10.1371/journal.pgen.1010136)
Supplement: Attachment — Submitted filename: response to reviewers and editor.docx [file pgen.1010136.s020.docx]

Additional typos are:

- Page 8 line 16- Microtubules instead microtubles
- Page 16 line 20 imaging instead imagng
- Page 42 line 9 occurred instead occured
- page 44 line 3 expressing instead expresing

These typos have been corrected.

- Fig. 2D is called before 2B and 2C, the order of figure 7 panels calls not right

Figure 2B is now mentioned in the results section before 2C and 2D, and we have switched the images in 2C and 2D so that they now match the text in Results. The figure legend has also been modified.

Switched images in Fig 7. Fig 7C and D are now Fig 7E and F. Made corrections to call outs in the text and in the figure legend

Reviewer #1: The revised manuscript includes new data that address the most critical referee comments and suggestions made during the second round of reviews. The manuscript is much stronger and more easily understood because of these revisions, and the data and conclusions regarding cohesin-dependent but cohesion-independent roles for cohesin in spindle assembly are quite convincing and interesting. My concerns have been adequately addressed.

No changes were made in response to reviewer 1.

Reviewer #2: The reviewers have addressed my concerns in this revised version. In my final readthrough I caught a few minor errors that I suggest correcting. Otherwise, I am supportive of publication.

- The section describing Figure 2 (starting on line 9) describes events happening in live movies and refers to the timing of events. However, some of the timestamps don’t match when comparing between panels. For example, page 8 line 11 states that microtubules assemble at a similar time after ovulation as control, but if you look at the timestamps in panels 2B and 2D, it does not seem similar (Metaphase I is at -3:20 in panel B, but at 2:00 in panel D). Similarly, the next sentence states that the amorphous cloud shrinks in similar timing, but lists two different timepoints (7:50 and -1:10). Why are these timestamps so different if the authors are claiming they are “similar”? It is possible that this timing difference is explained somewhere, but if so, I missed it. I think it would be useful to add some clarifying information to the figure legend, which is where I think most people would look for this type of information if they are confused.

This has been corrected. Metaphase I in Fig. 2B is -3.20 and in 2C it is now a similar -4.20. The previous stamps for 2C were based on a different time 0, which we failed to explain. We have added a statement to the legend that 0:00 is the time when the spindle makes full contact with the cortex.

- Figure S2A does not have a figure call-out in the results section.

Added a call-out to Figure S2A in the text

- I think that “S5C” on page 14 line 10 is supposed to be S5B. Also, in Figure S5 itself, I think the “B” needs to be moved up (it is placed below labels that should be part of the panel).

Fixed the call-out to S5B Fig

Moved up the “B” in S5 Fig

Reviewer #3: The authors have done a satisfactory job revising the manuscript. I have no further comments.

No changes were made in response to reviewer 3.

We noticed that there was no text call out for Fig. 2E so we added this to the results text.

We noticed incorrect call out sequence for Fig. 9D,E,F and changed the order of the text to correct this.

We noticed that rec-8 was not italicized in Fig. 5C and have corrected this.
